# Abnormal DNA methylation within HPA-axis genes years after paediatric critical illness

**DOI:** 10.1186/s13148-024-01640-y

**Published:** 2024-02-23

**Authors:** Grégoire Coppens, Ilse Vanhorebeek, Fabian Güiza, Inge Derese, Pieter J. Wouters, Arno Téblick, Karolijn Dulfer, Koen F. Joosten, Sascha C. Verbruggen, Greet Van den Berghe

**Affiliations:** 1 Clinical Division and Laboratory of Intensive Care Medicine, Department of Cellular and Molecular Medicine, KU Leuven, Herestraat 49, 3000 Leuven, Belgium; 2 Division of Paediatric Intensive Care Unit, Department of Neonatal and Paediatric ICU, Erasmus Medical Centre, Sophia Children’s Hospital, Rotterdam, The Netherlands

**Keywords:** Critical illness, Children, DNA methylation, HPA axis, Glucocorticoid, Long-term outcome

## Abstract

**Background:**

Critically ill children suffer from impaired physical/neurocognitive development 2 years later. Glucocorticoid treatment alters DNA methylation within the hypothalamus–pituitary–adrenal (HPA) axis which may impair normal brain development, cognition and behaviour. We tested the hypothesis that paediatric-intensive-care-unit (PICU) patients, sex- and age-dependently, show long-term abnormal DNA methylation within the HPA-axis layers, possibly aggravated by glucocorticoid treatment in the PICU, which may contribute to the long-term developmental impairments.

**Results:**

In a pre-planned secondary analysis of the multicentre PEPaNIC-RCT and its 2-year follow-up, we identified differentially methylated positions and differentially methylated regions within HPA-axis genes in buccal mucosa DNA from 818 former PICU patients 2 years after PICU admission (*n* = 608 no glucocorticoid treatment; *n* = 210 glucocorticoid treatment) versus 392 healthy children and assessed interaction with sex and age, role of glucocorticoid treatment in the PICU and associations with long-term developmental impairments. Adjusting for technical variation and baseline risk factors and correcting for multiple testing (false discovery rate < 0.05), former PICU patients showed abnormal DNA methylation of 26 CpG sites (within *CRHR1, POMC, MC2R, NR3C1, FKBP5, HSD11B1, SRD5A1, AKR1D1, DUSP1, TSC22D3* and *TNF*) and three DNA regions (within *AVP, TSC22D3* and *TNF*) that were mostly hypomethylated. These abnormalities were sex-independent and only partially age-dependent. Abnormal methylation of three CpG sites within *FKBP5* and one CpG site within *SRD5A1* and *AKR1D1* was partly attributable to glucocorticoid treatment during PICU stay. Finally, abnormal methylation within *FKBP5* and *AKR1D1* was most robustly associated with long-term impaired development.

**Conclusions:**

Two years after critical illness in children, abnormal methylation within HPA-axis genes was present, predominantly within *FKBP5* and *AKR1D1*, partly attributable to glucocorticoid treatment in the PICU, and explaining part of the long-term developmental impairments. These data call for caution regarding liberal glucocorticoid use in the PICU.

**Supplementary Information:**

The online version contains supplementary material available at 10.1186/s13148-024-01640-y.

## Background

Critical illness in children, which requires treatment in a paediatric intensive care unit (PICU), represents a form of severe physical stress, hallmarked by a whole range of (neuro)endocrine abnormalities [1–5]. A typical response to the severe stress of critical illness is an acute activation of the hypothalamus–pituitary–adrenal (HPA) axis [1, 6, 7]. Indeed, critically ill patients show an acute rise in total and free cortisol, lasting shorter in children than in adults [1, 8–11]. Unlike long assumed, the rise in cortisol is not driven by a rise in ACTH, as ACTH levels have been shown to be normal or low during critical illness [1, 10, 11]. Instead, the elevated cortisol levels appeared explained by low levels of its binding proteins corticosteroid-binding globulin and albumin, combined with suppressed breakdown of cortisol [1, 11]. Suppressed cortisol breakdown has been revealed by reduced expression and/or activity of the cortisol metabolising enzymes 11β-hydroxysteroid dehydrogenase (11βHSD) 2 and the A-ring reductases, and by strongly reduced cortisol plasma clearance during tracer infusion and after administration of a hydrocortisone bolus [1, 11].

Follow-up studies of children who needed PICU admission for a wide variety of surgical or medical reasons have shown that these children reveal high risk for important impairments in physical and neurocognitive development and behavioural problems years after the acute illness, also in the absence of pre-existing conditions known to affect or possibly affect development [12–17]. Other severe early-life adverse events, such as abuse or neglect occurring during several sensitive neurodevelopmental windows, have been associated with similar neurocognitive impairments and behavioural problems and with risk of psychiatric and metabolic diseases later in life [18–20]. Some of these associations appear to be sex- and/or age-specific [20].

Research has suggested that stress affects development of brain regions crucial for many aspects of cognition and behaviour through increased glucocorticoid signalling that, when prolonged or excessive, can induce abnormal DNA methylation within different levels of the HPA-axis and glucocorticoid signalling [21–24]. More specifically, such DNA-methylation changes have been reported for genes encoding corticotropin-releasing hormone (*CRH*) and its receptor *CRHR1*, arginine vasopressin (*AVP*) and its receptor *AVPR1b*, pro-opiomelanocortin (*POMC*), corticotropin receptor (*MC2R*), glucocorticoid receptor GRα (*NR3C1*) and for the GRα-regulated chaperone protein FK506 binding protein 51 (*FKBP5*) [23, 25–31]. In addition, altered methylation of genes encoding the cortisol metabolising enzymes 11βHSD1 (encoded by *HSD11B1*), 11βHSD2 (*HSD11B2*) and the A-ring reductases (*SRD5A1, SRD5A2, AKR1D1*) as documented in brain regions or peripheral tissues was suggested to be involved [32–38]. Previous work further suggested that GR binding to glucocorticoid-responsive elements (GREs) can induce changes in methylation of GR-target genes, such as dual-specificity phosphatase-1 (*DUSP1*), annexin A1 (*ANXA1*), pro-protein convertase-1 (PCSK1), glucocorticoid-induced leucine zipper (GILZ, encoded by *TSC22D3*) and tumour necrosis factor (TNF)-α (*TNF*) [39, 40].

In a large genome-wide analysis, we identified de novo alterations in leukocyte DNA methylation in critically ill children that rapidly arose after PICU admission and remained present until PICU discharge [41, 42]. In another large genome-wide analysis on buccal mucosa DNA obtained from those patients 2 years later, we further documented abnormal methylation as compared with healthy children, in pathways known to be important for physical/neurocognitive development [43]. In view of the striking similarities in long-term impairments in physical and neurocognitive development and behavioural problems after critical illness and other severe early-life adverse events and the suggested involvement of abnormal DNA methylation within the levels of the HPA-axis and glucocorticoid signalling in the long-term developmental impairments after such other early-life adverse events, we aimed to look more in-depth into this axis in a pre-planned targeted hypothesis-driven sub-analysis. In the present study, we thus addressed the hypothesis that infants, children and adolescents who have been admitted to the PICU, sex- and age-dependently, show long-term abnormal buccal mucosa DNA methylation within the different levels of the HPA-axis and glucocorticoid signalling, possibly aggravated by treatment with glucocorticoids in the PICU, which may contribute to the long-term physical and neurocognitive impairments and behavioural problems [12, 13].

## Methods

### Study population and buccal mucosal swab sampling

This study is a pre-planned secondary analysis of the multicentre PEPaNIC-RCT (registered at ClinicalTrials.gov NCT01536275, 2012-2015) and its 2-year follow-up study (2014–2018) [12, 44]. The PEPaNIC-RCT included 1440 consecutive critically ill children aged 0–17 years admitted to the PICUs of Leuven (Belgium), Rotterdam (The Netherlands) or Edmonton (Canada), who had an expected PICU stay of at least 24 h, were at risk of malnutrition, and did not meet any of the exclusion criteria [44]. All patients, who survived and for whom written informed consent was obtained, were eligible for a follow-up study 2 years after PICU admission, to assess physical, neurocognitive and emotional/behavioural development [12]. Healthy children with comparable sex and age distribution as the former PICU patients were included as controls. These were either siblings and relatives of the patients or unrelated children from the same geographical area, attempting to adjust as much as possible for genetic and socio-economic/environmental background [12]. The number of healthy children to be recruited as control group was based on power to detect relevant differences in developmental outcomes [12].

At the 2-year follow-up of the PEPaNIC patients, upon which time also the healthy children were assessed, buccal mucosal swabs (Isohelix, Cell Projects, Harrietsham, Kent, England) were collected following a standardised collection procedure [43]. Swabs were stored in a DNA stabilising solution (DSK kit, Isohelix) at − 80 °C until further processing. All former patients and healthy children from whom a buccal mucosal swab was available were eligible for this DNA methylation study (Additional file 1: Fig. A1). These were patients and healthy children who had been recruited in Leuven and Rotterdam, as the Edmonton centre did not participate in swab collection.

### Ethics

The institutional review boards at each participating site approved this study (Ethische Commissie Onderzoek UZ Leuven/KU Leuven: ML8052; Medische Ethische Toetsingscommissie Erasmus MC: NL49708.078). The study was performed in accordance with the 1964 Declaration of Helsinki and its amendments. Written informed consent was acquired from parents, legal guardians and/or the child if 18 years or older.

### DNA extraction and DNA methylation data processing

As previously described, DNA was extracted from all available buccal mucosal swabs from patients and healthy children (DDK DNA isolation kit, Isohelix) [43]. DNA concentrations were quantified with the Qubit® 3.0 fluorometer (Thermo Fisher Scientific, Waltham, MA). Two-hundred ng DNA was subsequently subjected to bisulfite-conversion with use of the EZ-96 DNA-methylation-Direct® Kit (Zymo Research, Irvine, CA). Bisulfite-converted DNA was profiled using the Infinium® HumanMethylation EPIC BeadChip (Illumina Inc., San Diego, CA), which interrogates 865,859 CpG sites. Analyses were performed blinded for former PICU patient (subgroup) versus healthy child status.

Data were processed using R statistical software version 4.0.2 using the LICMEpigenetics package (version 0.1.0) and the ilm10b4.hg19 annotation file using human genome 19 (hg19) as a reference was used to annotate the CpG sites [43]. This package contains R functions to exclude low-quality samples (not showing the typical bi-peak curve of the methylation value distribution) and probes (detection *p* value greater than 0.01 in 50% of the samples, or probes spanning single nucleotide polymorphisms), normalise the methylation data, adjust for batch effect and find differentially methylated positions and regions, as described below [45, 46]. Non-biological or technical variation due to experimental conditions was corrected for by including the first 30 principal components (PCs) of the technical control probes located on the Infinium® HumanMethylation EPIC BeadChip, excluding the negative control probes, as covariates in all multivariable linear regression models, according to the method developed by Lehne et al. [47].

### Selected genes

**Fig. 1:**
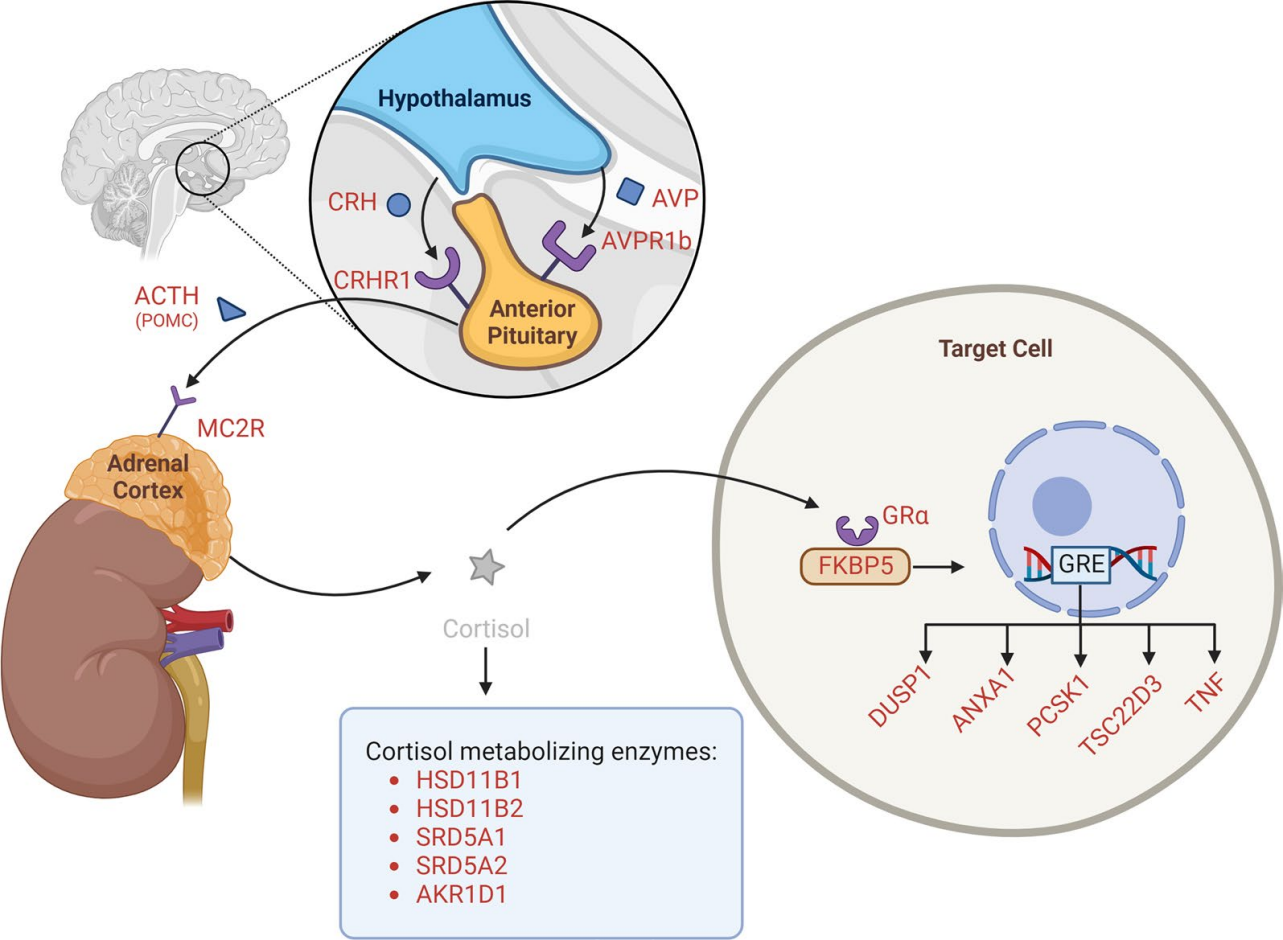
Schematic overview situating the studied genes within the different levels of the HPA-axis and glucocorticoid signalling. Please note that the DNA methylation status of the indicated genes was studied in buccal mucosa as a surrogate for the tissues in the figure. Created with BioRender.com. Abbreviations: GRE: glucocorticoid-responsive element

Figure 1 gives a schematic overview of the studied genes encoding key proteins within the different levels of the HPA-axis (*CRH*, *CRHR1*, *AVP*, *AVPR1b*, *POMC*, *MC2R*, *NR3C1*, *FKBP5, HSD11B1*, *HSD11B2, SRD5A1, SRD5A2,* and *AKR1D1*) and the selected GR-target genes (*DUSP1*, *ANXA1*, *PCSK1, TSC22D3*, and *TNF)*. Methylation of these genes was studied in buccal mucosa as a surrogate for the tissues indicated in the figure, which are not accessible for obvious reasons. The CpG sites within these genes analysed in this study are listed in Additional file 1: Table A1.

### Statistical analyses

Patient demographics and medical characteristics are reported as median and interquartile range or as number and percentage. Group comparisons were performed with the Chi-square test for categorical variables and with the Wilcoxon signed rank test for continuous variables.

#### DNA methylation changes between former PICU patients and healthy children

Differences in DNA methylation between former PICU patients and healthy children were assessed, correcting for multiple testing by applying a false discovery rate (FDR) of less than or equal to 0.05 as determined with the Benjamini–Hochberg procedure [48]. Such differences, if any, are to be considered the sum of differences evoked by the paediatric critical illness and intensive medical care and those that may have been present in the former patients prior to PICU admission.

First, all differentially methylated positions (DMPs) in former PICU patients as compared with the healthy children were identified. For each CpG site, methylation status was compared between former patients and healthy children with use of a multivariable linear model, using the limma framework, adjusting for baseline risk factors [age, centre, race, sex, geographical origin, history of malignancy and predefined syndrome (rationale provided in Additional file 1: Method A1, definition of syndrome provided in Additional file1: Method A2)] and adjusting for technical variation (batch effect) [47, 49]. Next, all differentially methylated regions (DMRs), i.e. regions within the DNA where groups of neighbouring CpG sites are differentially methylated, in former PICU patients as compared with the healthy children were identified with the DMRcate package [50]. A stepwise explanation of the DMRcate method with an illustrative example is provided in Additional file 1: Method A3 [43].

#### Interaction with sex and age

We further assessed, for the above identified DMPs in former PICU patients as compared with healthy children, whether there was any interaction with sex and/or the age ‘at exposure’. These analyses allow to investigate whether any difference between former PICU patients and healthy children would depend on sex or ‘age at exposure’. For the former PICU patients, age ‘at exposure’ was the age at admission to the PICU. For the healthy children, the age ‘at exposure’ was imputed by subtracting 2 years from their age at 2-year follow-up assessment [12]. Assessment of interactions was done by adding an interaction term in the multivariable linear models described above.

#### Role of glucocorticoid treatment during PICU stay

We next investigated to what extent glucocorticoid treatment during PICU stay may have played a role in bringing about or aggravating any of the identified DMPs 2 years after PICU admission. To this end, we performed multivariable analyses with the limma framework, among former PICU patients, with use of the methylation status of each of the CpG sites identified above as the dependent variables, comparing glucocorticoid treatment versus no glucocorticoid treatment in the PICU. We adjusted for baseline risk factors and technical variation as described above, and for length of PICU stay, admission diagnosis, severity of illness [Paediatric Index of Mortality 3 (PIM3) score, Paediatric Logistic Organ Dysfunction (PeLOD) score], randomisation to one of two nutritional strategies and risk of malnutrition [STRONGkids score] (rationale provided in Additional file 1: Method A1).

#### Association with physical and neurocognitive/behavioural development

For each of the above identified DMPs present in former PICU patients as compared with healthy children, we next assessed with use of multivariable linear models, whether methylation status in patients was associated with the physical (weight, height and head circumference) and neurocognitive/behavioural outcomes (executive functioning, emotional/behavioural problems, intelligence, visual motor integration, alertness and memory) that were impaired as compared with healthy children at 2-year follow-up (a detailed description of these outcome measures is provided in Additional file 1: Method A4) [12], adjusting for all above listed baseline risk factors and technical variation, with the addition of ‘linguistic origin’, since this is a known confounder for the functional outcome assessment (rationale for each risk factor provided in Additional file 1: Method A1). To assess robustness of these linear models, we first performed a tenfold cross-validation and computed the *p* values of the 10 test folds using Fisher’s method [51]. This process was repeated in 100 iterations. The percentage of iterations with a significant *p* value across the tenfold of the cross-validation (*α* ≤ 0.05) was calculated and visualised in a heatmap. Additionally, with use of the mean coefficient from the multivariable models, we assessed whether the abnormal methylation that we observed in former PICU patients was associated with either better (*B*) or harmful (*H*) outcome.

## Results

### Sample collection and quality assessment

Buccal mucosal swabs were collected from 821 patients and from 392 healthy children (Additional file 1: Fig. A1). Three patients needed to be excluded for further analysis, as DNA yield was insufficient for 1 patient, and 2 samples from patients showed deviation from the typical bi-peak curve of the methylation value distribution. Of the 818 patients remaining for the analyses, 210 had received glucocorticoid treatment during PICU stay. Participants’ demographics and medical characteristics are shown in Table 1.

**Table 1:** Demographics and medical characteristics of participants included in the 2-year epigenetic follow-up analysis

Demographics and medical characteristics of participants	Healthy children *N* = 392	PICU patients *N* = 818	*p* value^i^	PICU patients No GC *N* = 608	PICU patients GC *N* = 210	*p* value
*Demographics*						
Age at 2-year follow-up (years)—median (IQR)	3.8 (2.6–8.2)	3.4 (2.6–7.9)	0.96	3.2 (2.6–7.9)	3.6 (2.6–8.0)	0.31
Sex			0.19			0.10
Male—no (%)	212 (54.1)	475 (58.1)		343 (56.4)	132 (62.9)	
Female—no (%)	180 (45.9)	343 (41.9)		265 (43.6)	78 (37.1)	
Known non-Caucasian race^a^—no (%)	32 (8.2)	66 (8.1)	0.95	42 (6.9)	24 (11.4)	0.045
Known non-European origin^a^—no (%)	51 (13.0)	144 (17.6)	0.038	96 (15.8)	48 (22.9)	0.023
Known not exclusive Dutch or English language—no (%)	73 (18.6)	190 (23.2)	0.066	140 (23.0)	50 (23.8)	0.81
Socioeconomic status						
Educational level parents^b^			< 0.0001			0.20
Educational level 1	13 (3.3)	41 (5.0)		32 (5.3)	9 (4.3)	
Educational level 1.5	22 (5.6)	59 (7.2)		43 (7.1)	16 (7.6)	
Educational level 2	54 (13.8)	201 (24.6)		147 (24.2)	54 (25.7)	
Educational level 2.5	73 (18.6)	135 (16.5)		99 (16.3)	36 (17.1)	
Educational level 3	212 (54.1)	208 (25.4)		167 (27.5)	41 (19.5)	
Educational level unknown	18 (4.6)	174 (21.3)		120 (19.7)	54 (25.7)	
Occupational level parents^c^			< 0.0001			0.44
Occupational level 1	2 (0.5)	12 (1.5)		10 (1.6)	2 (1.0)	
Occupational level 1.5	23 (5.9)	75 (9.2)		58 (9.5)	17 (8.1)	
Occupational level 2	49 (12.5)	141 (17.2)		102 (16.8)	39 (18.6)	
Occupational level 2.5	29 (7.4)	79 (9.7)		63 (10.4)	16 (7.6)	
Occupational level 3	85 (21.7)	130 (15.9)		99 (16.3)	31 (14.8)	
Occupational level 3.5	41 (10.5)	54 (6.6)		40 (6.6)	14 (6.7)	
Occupational level 4	116 (29.6)	111 (13.6)		87 (14.3)	24 (11.4)	
Occupational level unknown	47 (12.0)	216 (26.4)		149 (24.5)	67 (31.9)	
*Medical characteristics*						
STRONGkids risk level^d^			–			0.30
Medium—no (%)	NA	736 (90.0)		551 (90.6)	185 (88.1)	
High—no (%)	NA	82 (10.0)		57 (9.4)	25 (11.9)	
PeLOD score, first 24h in PICU^e^—mean (SD)	NA	22 (12–32)	–	22 (12–32)	21 (11–31)	0.012
PIM3 score^f^—mean (SD)	NA	− 3.8 (− 4.4 to − 2.7))	–	− 3.9 (− 4.4 to − 2.9)	− 3.0 (− 4.3 to − 0.9)	< 0.0001
PIM3 probability of death^g^ (%)—mean (SD)	NA	2.3 (1.2–6.5)	–	2.0 (1.2–5.3)	4.6 (1.4–10.4)	< 0.0001
Randomisation to late-PN	NA	412 (50.4)	-	303 (49.8)	109 (51.9)	0.60
Diagnostic category			–			< 0.0001
Cardiac surgery—no (%)	NA	364 (44.5)		308 (50.7)	56 (26.7)	
Elective other surgery—no (%)	NA	116 (14.2)		85 (14.0)	31 (14.8)	
Urgent other surgery—no (%)	NA	142 (17.4)		96 (15.8)	46 (21.9)	
Medical diagnosis—no (%)	NA	196 (24.0)		119 (19.6)	77 (36.7)	
Malignancy—no (%)	0 (0.0)	39 (4.8)	< 0.0001	18 (3.0)	21 (10.0)	0.0001
Syndrome^h^—no (%)	4 (1.0)	168 (20.5)	< 0.0001	118 (19.4)	50 (23.8)	0.17

*GC* glucocorticoids, *IQR* interquartile range, *NA* not applicable, *no* number, *PeLOD* paediatric logistic organ dysfunction score, *PICU* paediatric intensive care unit, *PIM3* paediatric index of mortality 3 score, *PN* parenteral nutrition, *SD* standard deviation

^a^Participants were classified according to race and geographical origin by the investigators

^b^The education level is the average of the paternal and maternal educational level, and calculated based upon the 3-point scale subdivisions as made by the Algemene Directie Statistiek (Belgium; statbel.fgov.be/nl/) and the Centraal Bureau voor de Statistiek (The Netherlands; statline.cbs.nl): Low (= 1), middle (= 2) and high (= 3) educational level (see Additional file 1: Methods A5)

^c^The occupation level is the average of the paternal and maternal occupation level, which is calculated based upon the International Isco System 4-point scale for professions (see Additional file 1: Methods A5)

^d^Scores on the Screening Tool for Risk on Nutritional Status and Growth (STRONGkids) range from 0 to 5, with a score of 0 indicating a low risk of malnutrition, a score of 1 to 3 indicating medium risk and a score of 4 to 5 indicating high risk

^e^Paediatric Logistic Organ Dysfunction (PeLOD) scores range from 0 to 71, with higher scores indicating more severe illness

^f^Paediatric Index of Mortality 3 (PIM3) scores, with higher scores indicating a higher risk of mortality

^g^Paediatric Index of Mortality 3 (PIM3) probability of death

^h^A pre-randomisation syndrome or illness a priori defined as affecting or possibly affecting development (see Additional file 1: Methods A1)

^i^Chi-square test for categorical variables and Wilcoxon signed rank test for continuous variables was used to calculate *p* values

### DNA methylation differences between former PICU patients and healthy children

The adjusted comparison of the former PICU patients with the healthy children identified 26 DMPs within the HPA-axis genes. Their location within the genome, and the direction of change (hypo- or hypermethylated in former patients as compared with healthy children) are listed in Table 2. The absolute mean differences in DNA-methylation beta-values for the DMPs were 2.2% (SD 1.5%), ranging up to 5.5%, with mostly hypomethylation in patients (20/26 (76.9%), Table 2, Additional file 1: Table A1, Additional file 1: Fig. A2). The 26 DMPs in former PICU patients as compared with healthy children were located in 11 out of the 18 selected genes (61.1%), more specifically *CRHR1* (1/41 CpG sites (2.4%)), *POMC* (1/27 CpG sites (3.7%)), MC2R (1/20 CpG sites (5.0%)), *NR3C1* (3/89 CpG sites (3.4%)), *FKBP5* (7/51 CpG sites (13.7%)), *DUSP1* (1/33 CpG sites (3.0%)), *TSC22D3* (2/50 CpG sites (4.0%)), *TNF* (3/27 CpG sites (11.1%)), *TSC22D3* (1/50 CpG sites (2.0%)) and *HSD11B1* (2/25 CpG sites (8.0%)), *SRD5A1* (1/31 CpG sites (3.2%)), *AKR1D1* (4/15 CpG sites, (26.7%)).

**Table 2:** Abnormal DNA methylation within the HPA-axis

Gene	CpG site	Gene section^a,b^	Former PICU patients versus healthy children						Former PICU patients: GC versus No CG treatment
			Methylation status^c^	Log Fold Change [Confidence Interval]^d^	Absolute mean difference^e^	*p* value^f^			*p* value^f^
						Pt vs Ctrl^g^	Int. sex	Int. age	
CRHR1	cg15607306	Body	Hypo	− 0.066 [− 0.108 to − 0.023]	0.004	0.049	0.87	0.91	0.77
POMC	cg09672383	5’UTR	Hypo	− 0.082 [− 0.135 to − 0.03]	0.018	0.049	0.70	0.62	0.11
MC2R	cg26344168	Body	Hypo	− 0.07 [− 0.113 to − 0.027]	0.011	0.049	0.44	0.52	0.83
NR3C1	cg26720913	5’UTR/1stExon	Hypo	− 0.163 [− 0.265 to − 0.062]	0.037	0.049	0.46	0.27	0.14
	cg01967637	Promoter/5’UTR/1stExon	Hyper	0.1 [0.037 – 0.163]	0.02	0.049	0.33	0.84	0.080
	cg15910486	Promoter/5’UTR	Hyper	0.09 [0.039 – 0.141]	0.02	0.033	0.64	0.87	0.62
FKBP5	cg23416081	5’UTR	Hypo	− 0.292 [− 0.432 to − 0.152]	0.055	0.0052	0.54	0.48	0.017
	cg15929276	5’UTR	Hypo	− 0.252 [− 0.397 to − 0.107]	0.051	0.037	0.36	0.83	0.0045
	cg03591753	5’UTR	Hypo	− 0.132 [− 0.176 to − 0.088]	0.031	< 0.00001	0.58	0.0060	0.26
	cg20813374	Promoter/5’UTR	Hypo	− 0.126 [− 0.183 to − 0.07]	0.02	0.0019	0.91	0.055	0.60
	cg03546163	5’UTR	Hypo	− 0.178 [− 0.248 to − 0.108]	0.029	0.00012	0.97	0.00063	0.92
	cg01839003	5’UTR	Hypo	− 0.077 [− 0.125 to − 0.029]	0.007	0.049	0.37	0.11	0.036
	cg22363520	Body	Hypo	− 0.253 [− 0.338 to − 0.167]	0.017	< 0.00001	0.83	0.10	0.068
HSD11B1	cg06571187	Promoter/5’UTR	Hypo	− 0.108 [− 0.177 to − 0.039]	0.017	0.049	0.94	0.027	0.18
	cg14139038	Promoter/5’UTR	Hypo	− 0.078 [− 0.118 to − 0.039]	0.01	0.0098	0.42	0.0020	0.49
SRD5A1	cg22911074	Promoter	Hyper	0.081 [0.031 – 0.13]	0.01	0.049	0.72	0.90	0.018
AKR1D1	cg02118020	Body	Hyper	0.188 [0.067 – 0.31]	0.047	0.049	0.25	0.25	0.017
	cg05082563	Body	Hypo	− 0.127 [− 0.208 to − 0.046]	0.024	0.049	0.81	0.67	0.11
	cg27250318	Body	Hypo	− 0.181 [− 0.29 to − 0.071]	0.033	0.049	0.70	0.42	0.16
	cg09373725	Body	Hypo	− 0.098 [− 0.159 to − 0.038]	0.015	0.049	0.82	0.22	0.45
DUSP1	cg00593243	Body	Hyper	0.184 [0.071 – 0.297]	0.042	0.049	0.28	0.34	0.11
TSC22D3	cg26954928	Body	Hypo	− 0.099 [− 0.161 to − 0.037]	0.002	0.049	0.38	0.061	0.41
	cg11907074	Promoter/Body	Hyper	0.114 [0.052 – 0.176]	0.008	0.020	0.86	0.56	0.69
TNF	cg10650821	1stExon	Hypo	− 0.059 [− 0.09 to − 0.029]	0.014	0.012	0.82	0.45	0.17
	cg26729380	1stExon	Hypo	− 0.052 [− 0.085 to − 0.019]	0.01	0.049	0.75	0.80	0.66
	cg08553327	1stExon	Hypo	− 0.062 [− 0.099 to − 0.025]	0.01	0.049	0.89	0.49	0.28

*GC* glucocorticoid, *PICU* paediatric intensive care unit, *UTR* untranslated region, *Pt vs Ctrl* former PICU vs healthy control children, *Int*. interaction with

^a^A CpG site can be located within multiple genes or splice variants and thus can be situated within multiple gene sections

^b^Promoter is defined as 0 to 1500 base pairs upstream of the transcription start site

^c^The methylation status of the former PICU patients compared to Healthy control children. ‘Hypo’ refers to the former PICU patients showing less methylation in a given CpG site (hypomethylated) compared to the healthy controls. ‘Hyper’ refers to the former PICU patients showing more methylation in a given CpG site (hypermethylated)

^d^Log FC: Log fold change in M values between former PICU patients and healthy controls adjusted for risk factors. The 95% confidence interval is shown

^e^ The absolute mean difference is the (unadjusted) absolute difference between the mean beta value of a given CpG within the Former PICU patients and the mean beta value of a given CpG within the healthy control children

^f^ p values extracted from multivariable linear regression models, adjusted for baseline risk factors and technical variation. All p values come from separate models

^g^ Adjusted for multiple testing using a false discovery rate smaller than or equal to 0.05

Three DMRs were identified, located in the *AVP* gene (containing 4 CpG sites with a width of 665 bp), the *TSC22D3* gene (containing 8 CpG sites with a width of 438 bp) and the *TNF* gene (containing 12 CpG sites with a width of 146 bp) (Additional file 1: Table A2, Additional file 1: Fig. A3). The DMRs in *AVP* and *TNF* were hypomethylated, whereas the DMR in *TSC22D3* was hypermethylated in former PICU patients as compared with healthy children.

### Interaction with sex and age

**Fig. 2:**
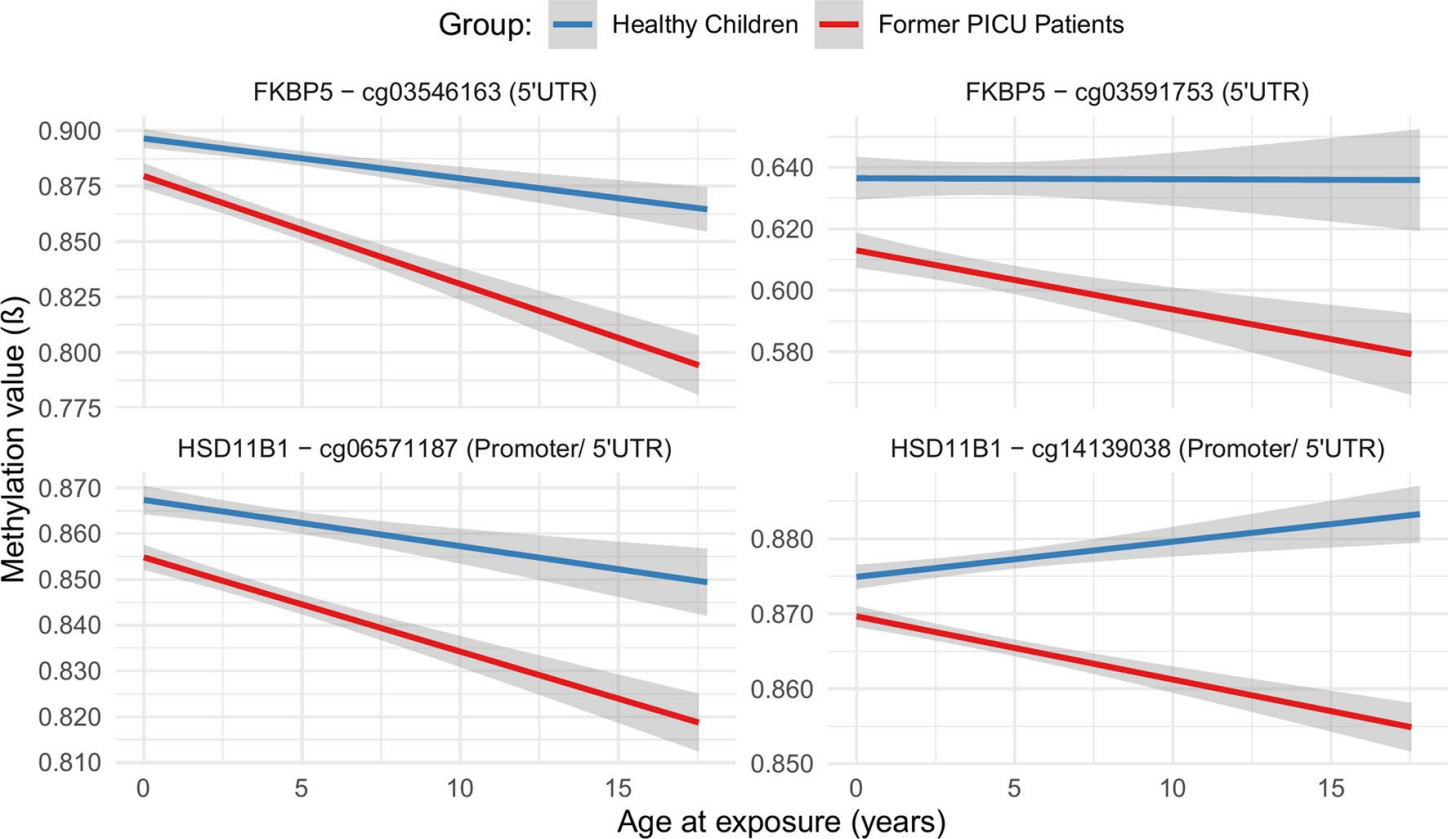
Interaction with age of abnormal DNA methylation in former PICU patients 2 years after critical illness vs healthy children. Methylation profiles are shown for each of the CpG sites that, adjusting for baseline risk factors and technical variation, showed a significant interaction between differential methylation in former PICU patients (*n* = 818) vs healthy children (*n* = 392) and ‘age at exposure’ (interaction p values obtained with multivariable linear regression analyses using the limma framework: cg03591753 *p* = 0.0060, cg03546163 *p* = 0.00063, cg06571187 *p* = 0.027, cg14139038 *p* = 0.0020). Univariate linear regression lines are drawn for methylation status of the CpG site in function of age at exposure, for former PICU patients (red) and healthy children (blue), where the shaded area represents the 95% confidence interval. Abbreviations: PICU: paediatric intensive care unit, UTR: untranslated region

The identified differences in DNA methylation between former PICU patients and healthy children were not sex-dependent (Table 2, Additional file 1: Table A3). Age ‘at exposure’ affected the methylation status of only 4 of the differentially methylated CpG sites (Table 2, Fig. 2, Additional file 1: Table A4). These CpG sites were located in the 5’UTR region of *FKBP5* (cg03591753 and cg03546163) and the promoter/5’UTR region (depending on transcription variant) of *HSD11B1* (cg06571187 and cg14139038). The degree of hypomethylation of these CpG sites in former PICU patients as compared with healthy children increased with age.

### Association between glucocorticoid treatment during PICU stay and DNA methylation differences

**Fig. 3:**
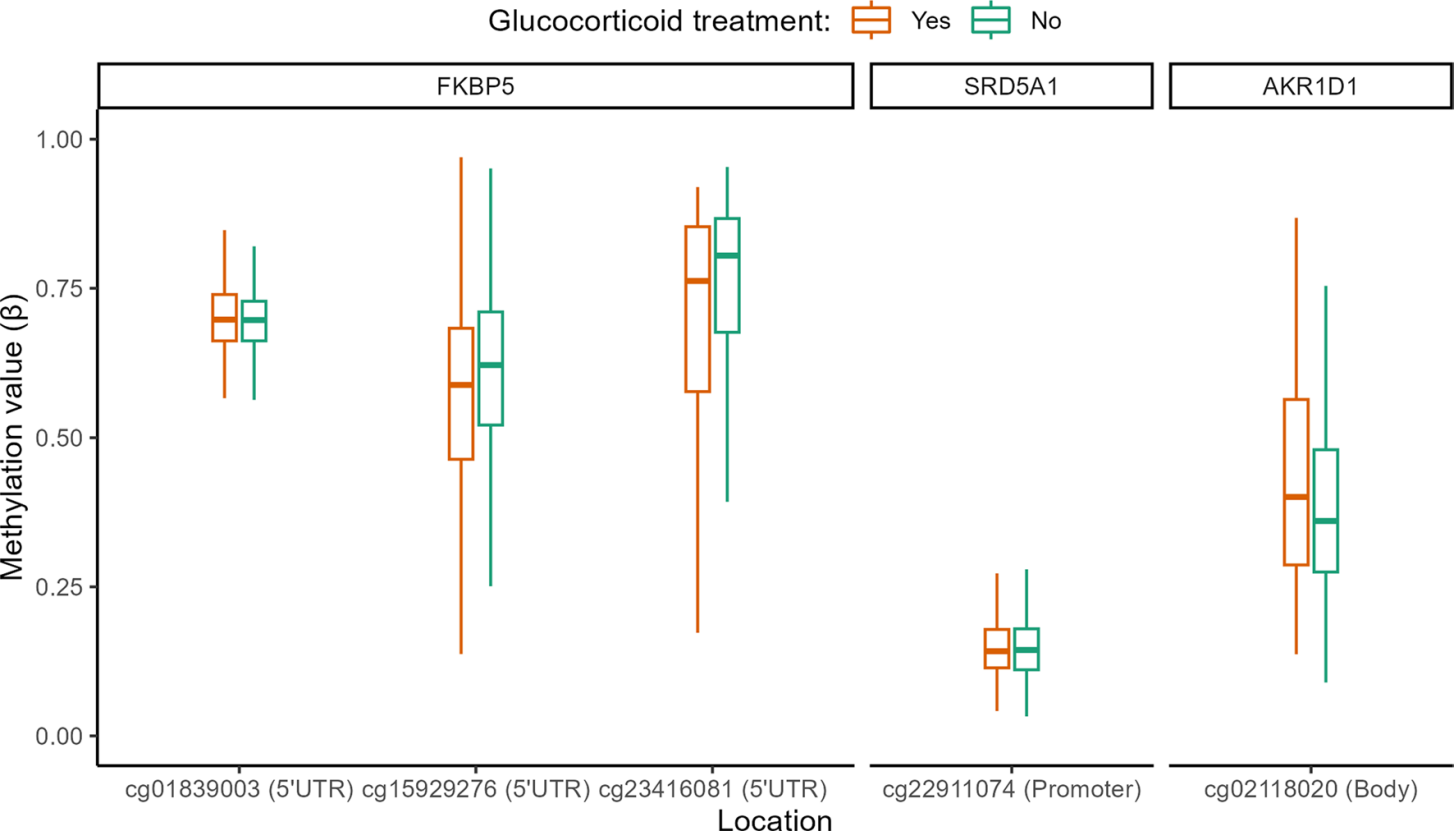
Role of glucocorticoid treatment in the PICU as a contributor to abnormal DNA methylation within HPA-axis genes 2 years later. The boxplots show a univariate presentation of the methylation status (*β* value) of the CpG sites of which abnormal methylation was aggravated by glucocorticoid treatment, as identified in multivariable analyses adjusting for baseline risk factors and technical variation (p values obtained with multivariable linear regression analyses using the limma framework: cg23416081 *p* = 0.017, cg15929276 *p* = 0.0045, cg01839003 *p* = 0.036, cg22911074 *p* = 0.018, cg02118020 *p* = 0.017). Former PICU patients who had received glucocorticoid treatment in the PICU (*n* = 210) are depicted in orange and those who had not (*n* = 608) are depicted in green. The central lines of the boxplots depict the medians, the boxes the interquartile ranges, and the whiskers are drawn to the furthest point within 1.5 times the interquartile range from the box. Abbreviations: UTR: untranslated region

We observed that three of the identified DMPs within *FKPB5* (cg23416081, cg15929276 and cg01839003), one DMP within *SRD5A1* (cg22911074) and one DMP in *AKR1D1* (cg02118020) appeared to be aggravated by glucocorticoid treatment given during PICU stay (Table 2, Fig. 3, Additional file 1: Table A5). The absolute differences in DNA methylation beta-values for these CpG sites, comparing former patients who had received glucocorticoid treatment with those who had not, were 3.1% (SD 2.3%), ranging up to 5.2%.

### Association between altered DNA methylation and impaired physical and neurocognitive/behavioural development

**Fig. 4:**
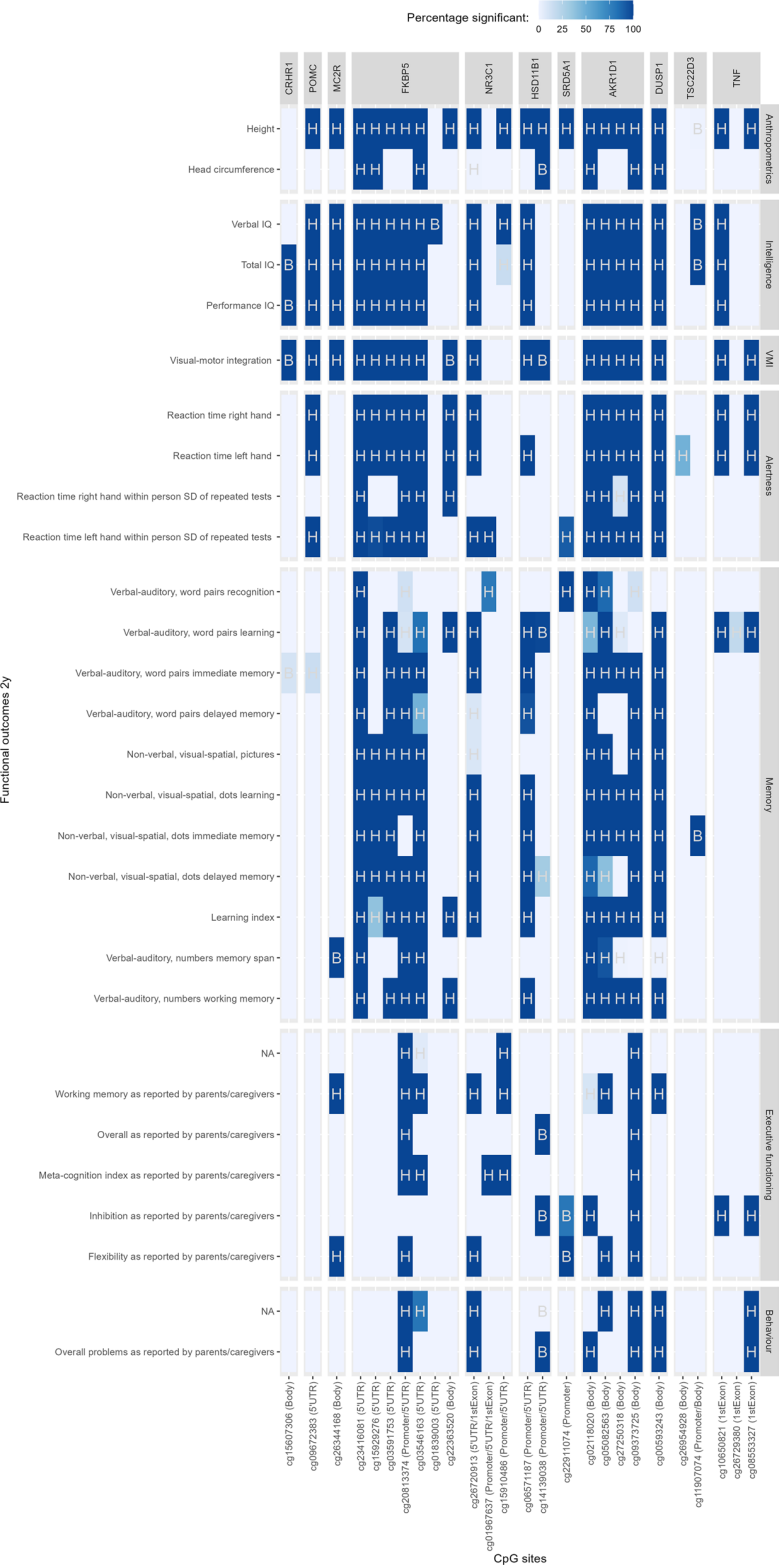
Association of long-term abnormal methylation within HPA-axis genes in former PICU patients with impaired long-term developmental outcomes. This heatmap summarises the results of the multivariable linear regression analyses assessing associations between abnormal methylation within HPA-axis genes 2 years after critical illness and developmental outcomes of former PICU patients evaluated at that time (*n* = 713), adjusted for baseline risk factors and technical variation. Each box shows the percentage of the 100 iterations for which the p values across the 10 test folds (computed with Fisher’s method [51]) are significant (*p* < 0.05) for a given CpG site (*X* axis) and outcome (*Y* axis). Darker colour intensity indicates a larger percentage of iterations being significant. For each CpG site, the location within the respective gene is also indicated (due to multiple splice variants, multiple gene locations are possible). The labels within the boxes indicate whether the observed abnormal methylation correlates with a better score for the long-term outcome (benefit ‘B’), or with a worse score (harm ‘H’). Abbreviations: IQ: intelligence quotient, SD: standard deviation, UTR: untranslated region, VMI: visual motor integration

The statistical association of DNA methylation differences between former PICU patients and healthy children with the impaired long-term physical growth and neurocognitive/behavioural functioning is visualised as a heatmap in Fig. 4. In the heatmap, we counted 325 significant associations of which 291/325 (89.5%) were found to be robust, being significant in 100% of the repetitions. Most associations were found for hypomethylation of CpG sites within the promoter/5’UTR of *FKBP5* in former patients, which statistically associated with more impaired physical growth, intelligence, visual motor integration, alertness and memory, and to a lesser extent with executive functioning and behavioural problems. Associations with impairments in the same domains were also found for abnormal methylation within the gene body of *AKR1D1*. For the other genes, mostly more discrete associations of abnormal methylation with more developmental impairments were observed, except for abnormal methylation within *CRHR1* and *TSC22D3*, which appeared to associate with less pronounced impairments in intelligence and/or VMI.

## Discussion

Two years after admission to the PICU, children who had been critically ill revealed abnormal DNA methylation within several genes of the HPA-axis. This was the case for genes encoding the CRH receptor, the ACTH precursor POMC and the ACTH receptor, the glucocorticoid receptor GRα and its co-chaperone FKBP5, several cortisol metabolising enzymes (11ß-HSD1, 5α-reductase and 5β-reductase) and three GR-regulated proteins (DUSP1, GILZ and TNFα). The most extensive changes were observed for the *FKBP5* and *AKR1D1* genes which were also substantially aggravated by glucocorticoid treatment in the PICU. Most abnormal DNA methylation in former patients was hypomethylation, was sex- and age-independent, and found to be independently associated with impaired physical and neurocognitive development.

Several critical illness- and treatment-related factors could be potential drivers for altered DNA methylation. Examples are hyperglycaemia and insulin resistance [52–56], neuroendocrine abnormalities [1–5, 57–59] and exposure to endocrine-disrupting plasticisers leaching from indwelling medical devices [60–62]. Also, as critically ill patients are unable to eat, artificial feeding is often provided [63]. Feeding management of critically ill children has been shown to affect the DNA methylome up until PICU discharge [41, 42]. Since prolonged or excessive stress can induce abnormal DNA methylation within different levels of the HPA-axis and glucocorticoid signalling in the context of other adverse early-life exposures [21–24, 59], the development of such abnormalities after paediatric critical illness seemed plausible. Our present findings are indeed in line with those from studies on the impact of other forms of early-life stress which revealed mostly hypermethylation in *NR3C1* and hypomethylation in *FKBP5* within the gene body (intron 7) and the promotor [64, 65]. Via research performed in humans in combination with experiments in cells, it has previously been shown that stress reduces methylation in the *FKBP5* gene, interestingly at a CpG site which we also found to be hypomethylated, whereby *FKBP5* expression was found to be upregulated [66]. Of importance in the context of critical illness, a study in mice revealed that lipopolysaccharide injection increased *FKBP5* expression in the hippocampus, driving increased neuroinflammation [67]. Whether the stress-induced rise in endogenous glucocorticoids mediated this effect is currently unclear. However, in patients with Cushing syndrome, it has been shown that long standing excessive endogenous hypercortisolism induces hypomethylation in *FKBP5*, explaining their long-lasting psychopathological sequelae [24]. In experimental models, also exogenous hypercortisolism has shown to reduce DNA methylation in *FKBP5* in neuronal cells, and in vivo this was associated with increased *FKBP5* expression across several brain regions [68]. These data provided a plausible explanation for our finding that hypomethylation in the *FKBP5* gene was aggravated in patients who 2 years earlier had been treated with glucocorticoids in the PICU, taking into account potential confounders affecting the need of glucocorticoid treatment. It currently remains unclear what brings about the hypomethylation, though downregulated DNA methyltransferase-1 and cleavage of the DNA backbone directly by the GRα have been suggested to play a role [39, 69–71]. Some of the differentially methylated CpG sites that we found within *NR3C1* and *FKBP5* have also been described with other forms of stress. For example, cg15910486 within the promotor/5’UTR of *NR3C1* was reported to be hypermethylated in children who experienced early-life adverse events; cg23416081 and cg15929276 located within the 5’UTR region of *FKBP5* have previously been shown to alter cortisol reactivity and behaviour in children; cg20813374 located in the promotor/5’UTR of *FKBP5* is a known stress-related epigenetic signature previously associated with myocardial infarction and inflammation; and cg03546163 in the 5’UTR of *FKBP5* was shown to be differentially methylated in the context of Cushing’s syndrome [24, 65, 66, 72].

Also glucocorticoid metabolism can be altered by early-life stress [33]. We here found that genes encoding cortisol metabolising enzymes were differently methylated in former PICU patients, more specifically for 5α- and 5β-reductase and 11β-HSD1. These changes could again be partly explained by glucocorticoid treatment during PICU stay years before. The methylation differences within *HSD11B1*, *SRD5A1* and *AKR1D1* may result in altered systemic cortisol availability and hereby affect development as was suggested by our functional outcome analysis. The *HSD11B1* hypomethylation we observed in former patients appeared to some extent protective against rather than contributing to some of the developmental impairments. However, a strong harmful association was found between most functional outcome measures and the abnormal DNA methylation in *AKRD1,* which encodes the 5β-reductase enzyme, an association that has not been reported before.

We also observed altered DNA methylation in former patients within the genes encoding CRH receptor, ACTH and its receptor MC2R, and three GR-regulated proteins, DUSP1, GILZ and TNFα, which has also been reported for other conditions of early-life stress [26, 28–30, 39, 40].

The observed differences in DNA methylation in former PICU patients as compared with healthy children were largely age- and sex-independent. However, we did find that the vulnerability to the illness-induced hypomethylation of 2 CpG sites within *FKBP5* and 2 CpG sites within *HSD11B1* was more pronounced with higher age at exposure. It has previously been shown that neonates and young infants are indeed relatively stress hyporesponsive, interpreted as a crucial protection against glucocorticoid-induced harmful effects on the developing brain [20, 73]. Higher vulnerability to illness-induced DNA-methylation changes in older than in younger children, in particular from age of adrenarche onwards and into puberty, has previously been shown by our group [74].

This study has some strengths and weaknesses to highlight. The large sample size and the multicentre, prospective study design with predefined long-term assessments of former PICU patients and healthy children were strengths. In addition, our methodology applying tenfold cross-validation over 100 iterations reduced the odds of findings by chance and reduced the impact of outliers. Our study also has some limitations. First, we have studied DNA methylation in buccal mucosa, whereas the HPA-axis links well-defined brain structures with the adrenal cortex via hormonal regulation. Studying the DNA-methylation markers of stress is obviously not possible in the physiological tissues and organs where it exerts its effects through the HPA-axis. It was thus for pragmatic reasons that we analysed buccal mucosa, as these epithelial cells are accessible to clinical research. These cells are also exposed to stress hormones, but we do not know whether the methylation changes observed in these cells reflect those supposed to occur in the HPA-axis itself. This difficulty has been encountered in other studies, where blood cells or buccal cells have been used as proxies to physiological tissues [23–25, 29, 31, 40–43, 66]. Second, due to lack of a sample before PICU admission, we cannot discriminate between pre-existing abnormal methylation and abnormal methylation induced by the critical illness. Third, although we preferentially recruited siblings and relatives of the patients to the control group, extended with unrelated children from the same geographical area and adjusted as much as possible for baseline risk factors, we cannot exclude residual confounding by genetic background and environment. Fourth, the treatment with glucocorticoids during PICU stay had not been randomised, arguing for caution when interpreting these results. Indeed, specific conditions triggering the need for glucocorticoid treatment theoretically may confound these results. However, with the extensive adjustment for risk factors which also included type, severity and duration of illness we aimed to reduce as much as possible the impact of this limitation. Nevertheless, we cannot exclude that there may be some residual unmeasured confounding in these and the other multivariable analyses. Finally, we were not able to assess what effect the abnormal DNA methylation might have on gene transcription, nor on cortisol or ACTH levels, as we did not have the samples for these analyses. However, differential methylation in the order of magnitude as observed in the present study was associated with differential gene expression in our earlier study on DNA methylation in muscle of adult critically ill patients and controls [75].

## Conclusions

Two years after critical illness in children, buccal mucosa DNA revealed abnormal methylation of CpG sites within genes of the HPA-axis, most extensively within the *FKBP5* and *AKR1D1* genes, which occurred largely independent of sex and age. In addition, glucocorticoid treatment while in the PICU was found to be associated with an aggravation of the methylation changes in *FKBP5* and *AKR1D1* detected 2 years later. The observed abnormal DNA methylation within these genes in former PICU patients statistically explained part of the long-term physical and neurocognitive developmental impairments. These findings call for attention regarding safety of a liberal glucocorticoid use in the PICU in the absence of strong underlying evidence of benefit.

### Supplementary Information

**Additional file 1.** Compiled file with all Additional information: Additional Methods describing the motivation of risk factors adjusted for in multivariable analyses, the definition of ‘Syndrome’ and a stepwise explanation of the DMRcate method for the identification of differentially methylated DNA regions, and detailed description of the outcome measures evaluated at the PEPaNIC 2-year follow-up; Additional Figures showing the CONSORT diagram of study participants, univariate boxplots of the methylation status of differentially methylated positions in former PICU patients as compared with matched healthy children, and univariate boxplots of the methylation status of the CpG sites within the regions identified as differentially methylated between former PICU patients and matched healthy children; and Additional Tables reporting on the DMP and DMR analyses for former PICU patients versus healthy children, interaction of differential methylation in former PICU patients versus healthy children with sex and age at exposure, and the analyses of differential methylation between former PICU patients who received glucocorticoids during their stay in the PICU versus those who did not.

## Data Availability

Data sharing is offered under the format of collaborative projects. Proposals can be directed to the corresponding author.

## Abbreviations

11βHSD: 11β-Hydroxysteroid dehydrogenase
ANXA1: Annexin A1
AVP: Arginine vasopressin
AVPR1b: Arginine vasopressin receptor 1b
CRH: Corticotropin-releasing hormone
CRHR1: Corticotropin-releasing hormone receptor 1
DMP: Differentially methylated position
DMR: Differentially methylated region
DUSP1: Dual-specificity phosphatase-1
FDR: False discovery rate
FKBP5: FK506 binding protein 51
GILZ: Glucocorticoid-induced leucine zipper
GRE: Glucocorticoid-responsive element
ICU: Intensive care unit
MC2R: Corticotropin receptor
NR3C1: Glucocorticoid receptor GRα
PCSK1: Pro-protein convertase-1
PeLOD: Paediatric logistic organ dysfunction score
PEPaNIC: Paediatric Early versus Late Parenteral Nutrition in Intensive Care Unit
PICU: Paediatric intensive care unit
PIM3: Paediatric index of mortality 3 score
POMC: Pro-opiomelanocortin
RCT: Randomised controlled trial
STRONGkids: Screening Tool for Risk on Nutritional Status and Growth for kids
TNF: Tumour necrosis factor-α
UTR: Untranslated region
