# Supplementary material for: Abnormal DNA methylation within HPA-axis genes years after paediatric critical illness
Source: Clin Epigenetics. 2024 Feb 23;16:31. doi: 10.1186/s13148-024-01640-y (PMC10893716; doi:10.1186/s13148-024-01640-y)
Supplement: Supplementary file 1 — Additional file 1. Compiled file with all Additional information: Additional Methods describing the motivation of risk factors adjusted for in multivariable analyses, the definition of ‘Syndrome’ and a stepwise explanation of the DMRcate method for the identification of differentially methylated DNA regions, and detailed description of the outcome measures evaluated at the PEPaNIC 2-year follow-up; Additional Figures showing the CONSORT diagram of study participants, univariate boxplots of the methylation status of differentially methylated positions in former PICU patients as compared with matched healthy children, and univariate boxplots of the methylation status of the CpG sites within the regions identified as differentially methylated between former PICU patients and matched healthy children; and Additional Tables reporting on the DMP and DMR analyses for former PICU patients versus healthy children, interaction of differential methylation in former PICU patients versus healthy children with sex and age at exposure, and the analyses of differential methylation between former PICU patients who received glucocorticoids during their stay in the PICU versus those who did not. [file 13148_2024_1640_MOESM1_ESM.docx]

ADDITIONAL FILE 1

**Abnormal DNA-methylation within HPA-axis genes years after paediatric critical illness**

Grégoire Coppens, M.D.^1^*, Ilse Vanhorebeek, M.Eng., Ph.D.^1^*, Fabian Güiza, M.Eng., Ph.D.^1^, Inge Derese B.Sc.^1^, Pieter J Wouters, M.Sc.^1^, Arno Téblick, M.D., Ph.D. ^1^, Karolijn Dulfer, M. Psych., Ph.D.^2^, Koen F. Joosten, M.D., Ph.D.^2^, Sascha C. Verbruggen, M.D., Ph.D.^2^, Greet Van den Berghe, M.D., Ph.D.^1^

* contributed equally

^1^ Clinical Division and Laboratory of Intensive Care Medicine, Department of Cellular and Molecular Medicine, KU Leuven, Leuven, Belgium; ^2^ Intensive Care Unit, Department of Paediatrics and Paediatric Surgery, Erasmus Medical Centre, Sophia Children’s Hospital, Rotterdam, The Netherlands.

**Address correspondence to:** Greet Van den Berghe, Clinical Division and Laboratory of Intensive Care Medicine, KU Leuven, Herestraat 49, B-3000 Leuven, Belgium. Phone: 32-16-34-40-21; Fax: 32-16-34-40-15; Email: greet.vandenberghe@med.kuleuven.be; ORCID: 0000-0002-5320-1362

**TABLE OF CONTENTS**

**Method A1:** Motivation of risk factors adjusted for in multivariable analyses.

**Method A2:** Definition of ‘syndrome’

**Method A3:** A stepwise explanation of the DMRcate method with an illustrative example

**Method A4:** Detailed description of outcome measures evaluated at PEPaNIC 2-year follow-up

**Method A5:** Definition of educational and occupational level of parents

**Figure A1:** CONSORT diagram of study participants

**Figure A2:** Univariate boxplots of the methylation status of differentially methylated positions in former PICU patients as compared with matched healthy children

**Figure A3:** Univariate boxplots of the methylation status of the CpG sites within the regions identified as differentially methylated between former PICU patients and matched healthy children

**Table A1:** DMP analysis – Former PICU patients vs Healthy children

**Table A2:** DMR analysis – Former PICU patients vs Healthy children

**Table A3:** DMP analysis – Former PICU patients vs Healthy children: Interaction with sex

**Table A4:** DMP analysis – Former PICU patients vs Healthy children: Interaction with age at exposure

**Table A5:** DMP analysis – Former PICU patients who received GC treatment vs those who did not

**References**

**Method A1: Motivation of risk factors adjusted for in multivariable analyses**

***Identification of differential DNA methylation in former PICU patients as compared with healthy children***

*- Age*: DNA methylation is age-dependent [1].

*- Sex*: DNA methylation is sex-dependent [2].

*- Treatment centre*: Takes into account slightly different protocols during the PEPaNIC study.

*- Race and geographical origin*: Race and regional origin can affect DNA methylation [3-5].

*- History of malignancy*: Altered DNA methylation is central to oncogenesis in many paediatric cancers [6,7].

*- Predefined syndrome*: Altered DNA methylation is involved in several syndromes [8-10].

***Role of glucocorticoid treatment during PICU stay in abnormal DNA methylation two years later***

- *Baseline risk factors* as described above.

- *Length of PICU stay*: Duration of PICU stay may be an important confounder in associations regarding glucocorticoid treatment.

- *Admission diagnosis and severity of illness* (Paediatric Index of Mortality 3 score, Paediatric Logistic Organ Dysfunction score): Glucocorticoid treatment may be required for specific critical illnesses/certain conditions.

- *Randomisation to one of two nutritional strategies and risk of malnutrition* (STRONGkids score): Inadequate nutrition can induce changes in the DNA methylome [11-14].

***Association of abnormal DNA methylation with physical and neurocognitive/behavioural development***

- *Age*: Physical growth and performance on neurocognitive developmental tests and vulnerability to adverse exposures are age-dependent.

- *Sex*: There is early and pervasive sexual differentiation in neurocognitive measures [15,16].

- *Treatment centre*: Takes into account slightly different protocols during the PEPaNIC study.

- *Race and geographical origin*: *Race and geographical origin* capture the ethnical and regional differences in the frequency of consanguinity, which might adversely affect cognitive and physical development [17,18]. Furthermore, associations with neurocognitive function have also been observed in the absence of consanguinity [5].

- *Linguistic origin*: May affect the extent of understanding instructions or fluency to perform tests.

- *History of malignancy*: Patients with a *history of malignancy* may show declines in both global and specific areas of neurocognitive functioning as well as in physical functional performance [19-22].

- *Predefined syndrome*: This was defined as a prerandomisation syndrome or illness *a priori* defined as affecting or possibly affecting neurocognitive development and hence needs to be adjusted for.

- Length of PICU stay: Duration of adverse exposures may affect development.

- *Admission diagnosis and severity of illness* (Paediatric Index of Mortality 3 score, Paediatric Logistic Organ Dysfunction score): The vulnerability of brain and physical development to environmental disturbances justifies the adjustments for type and severity of illness.

- *Randomisation to one of two nutritional strategies and risk of malnutrition* (STRONGkids score): Inadequate nutrition (both underfeeding and excessive nutrition) has been related with worse cognitive performance, behavioural problems, and impaired physical development [23-25].

**Method A2: Definition of ‘syndrome’**

A prerandomisation syndrome or illness *a priori* defined as affecting or possibly affecting neurocognitive development, and which is subdivided in the following categories:

Genetically confirmed syndrome or pathogenic chromosomal abnormality

Clearly defined syndrome, association or malformation without (identified) genetic aberration

Polymalformative syndrome of unknown aetiology

Clear auditory or visual impairment without specified syndrome

Congenital hypothyroidism due to thyroid agenesis

Brain tumour or tumour with intracranial metastatic disease

Paedopsychiatric disorder (e.g. autism spectrum disorder, (treatment for) attention deficit hyperactivity disorder)

Severe medical disorder, not primarily neurologic, but suspected to alter psychomotor and/or mental performance

Severe neonatal problem (e.g. severe asphyxia)

Severe craniocerebral trauma or near-drowning

Severe infectious encephalitis or drug-induced encephalopathy

Infectious meningitis, encephalitis or Guillain-Barré

Resuscitation and/or need for extracorporeal membrane oxygenation prior to randomisation

Severe convulsions or stroke prior to randomisation.

**Method A3: A stepwise explanation of the DMRcate method with an illustrative example**

Due to the complexity of the ‘dmrcate’ method, we here illustrate this technique in a simplified stepwise manner, using an example. The data used in this theoretical example are for illustrative purposes only.

Step 1: We take the squared moderated t-statistic from the multivariable linear regression models that were used to identify the DMPs.

Step 2: We calculate the kernel estimate for every location in the DNA that contains a CpG site. A kernel estimate is the weighted sum of all the moderated t² statistics in a given chromosome. Weights are defined by the distance from the location of interest using gaussian smoothing. Weights decrease with increasing distance to the CpG site of interest.

As demonstrated in the figure below, the kernel estimate of CpG 6 can be computed as follows (underlined: the t-statistic, **bold:** the kernel weight based on the distance from the CpG 6):

Weighted sum: 4.55***0.01** + 3.22***0.05** + … + 2.13***0.29** + 7.37***1.00** + 6.12***0.18 +** … + 5.89***0.08 =** 10.56

Step 3: Calculate the p-value of every kernel estimate against a null hypothesis of no methylation difference. In the example below, statistically significant kernel estimates are denoted in green.

Step 4: Agglomerate statistically significant kernel estimates that are not further then 1000 bp (λ) apart.

This stepwise explanation is a simplified version of the original method, for more information on how the weights of the kernel estimates are calculated or how the p-values are calculated, we refer to the work from Peters et al, 2015: <https://doi.org/10.1186/1756-8935-8-6> [26].


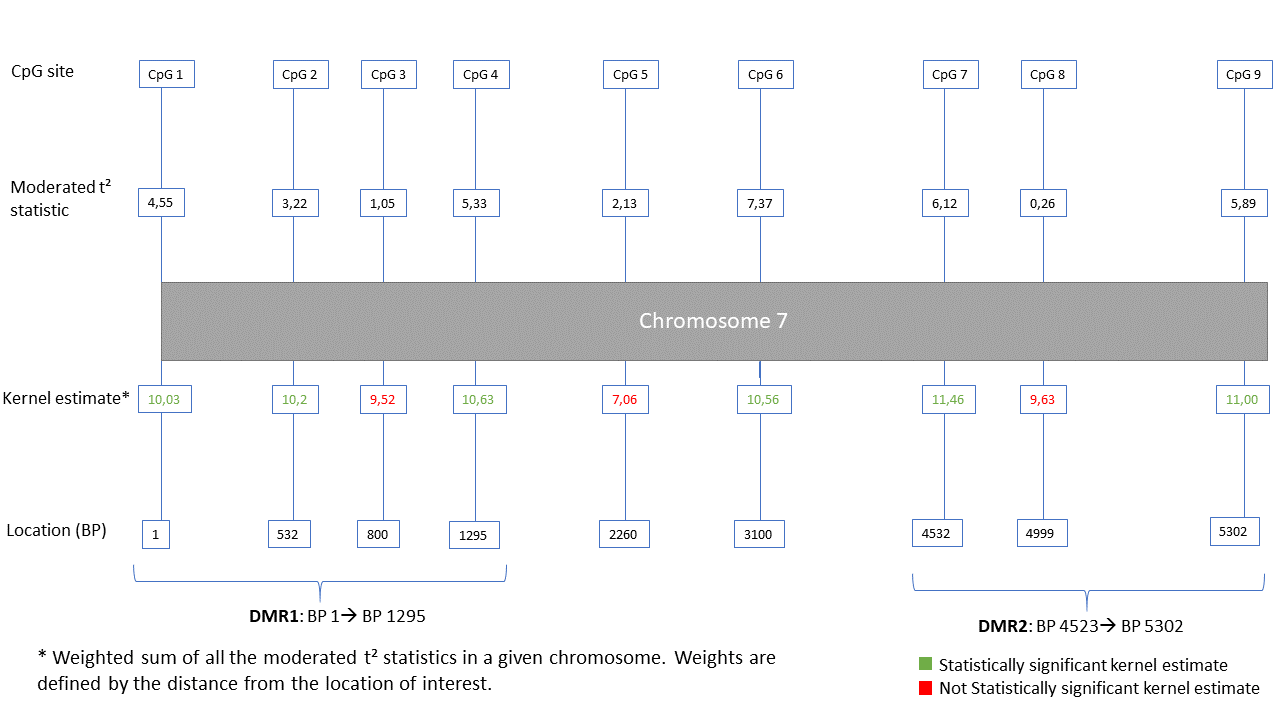


**Method A4: Detailed description of outcome measures evaluated at PEPaNIC 2-year follow-up**

***Medical assessment***

Height (in cm) and head circumference (in cm) were measured at the beginning of the follow-up visit.

***Neurocognitive testing***

A broad range of neurocognitive functions, including general intellectual functioning, visual-motor integration, attention, motor coordination, inhibitory control and cognitive flexibility, verbal and visual-spatial learning, and memory were evaluated, as previously reported [27].

*Parent-reported outcomes*

Executive functioning was measured with the Behaviour Rating Inventory of Executive Function (BRIEF-P 2.5- 5 years, BRIEF 6-18 years), filled out by the parents/caregivers of the child. Overlapping scales and indices of both questionnaires (Inhibition, Flexibility, Working Memory, Planning and Organisation, Meta-cognition) and a Total Score were analysed (T-scores, with mean 50 and SD 10) [28,29].

Emotional and behavioural problems were assessed by the parent/caregiver with the Child Behaviour Checklist (CBCL 1.5-5 years or CBCL 6-18 years) [30,31]. Internalising, externalising, and total problems were analysed (T-scores, with mean 50 and SD 10) [30,31].

*Intelligence*

General intellectual ability was assessed with use of age-appropriate versions of the Wechsler Intelligence Quotient (IQ) tests. The Wechsler Preschool and Primary Scale of Intelligence (WPPSI-III-NL) [32] was used for children aged 2.5 years – 5 years 11 months (one version for age range 2 years 6 months – 3 years 11 months, and another version for age range 4 years – 5 years 11 months), the Wechsler Intelligence Scale for Children (WISC-III-NL) [33] was used for children aged 6 years – 16 years 11 months, and the Wechsler Adult Intelligence Scale (WAIS-IV-NL [34]) for adolescents who were 17 years or older. For all these tests Total IQ, Verbal IQ, and Performance IQ scores (Test-mean 100, SD 15) were computed.

*Visual-motor integration*

We used the Beery Developmental Test of Visual-Motor Integration, 6th Edition (VMI) to assess the ability to integrate their visual and motor functions (total Scaled Score, Test-mean 10, SD 3). This involves eye-hand coordination [35].

*Alertness*

Children 4 years and older performed the ANT-Baseline Speed (BS) task of the validated Amsterdam Neuropsychological Tasks (ANT) program [36]. The ANT is a computer-aided assessment battery of reaction time (RT) tasks that allows for the systematic evaluation of information processing capacities. The ANT-BS evaluated alertness by measuring simple RT to visual stimuli (mean RT and SD of RT were obtained for the right and left hand separately).

*Memory*

Auditory/verbal memory and Visual-spatial/non-verbal memory were assessed with use of four tests from the Children’s Memory Scale (CMS) for children between 5 and 16 years 11 months [37].

As to verbal memory, CMS-Numbers assessed short-term verbal memory span (forward digit recall) and verbal working memory load (backward digit recall). The CMS-Word Pairs (recall a list of word pairs) assessed short-term and long-term verbal memory, and recognition.

As to non-verbal memory, CMS-Picture Locations (remembering and recall of pictures in various locations) assessed immediate visual memory. CMS-Dot Locations (remembering and recall of the location of dots) assessed immediate and delayed visual memory.

For CMS-Numbers, raw scores for verbal memory span, CMS-numbers forward, and verbal working memory load, CMS-numbers backward were reported. For CMS-Word Pairs, CMS-Picture Locations, and CMS-Dot Locations, proportional scores were analysed (proportion of correct responses ranging from 0 to 1, with higher scores reflecting better performance).

The CMS-Learning index is a standardised score of the sum of the three learning trials of the CMS-Word Pairs and the learning trial of the CMS-Dot Locations subtests. The range of the score is 50-150, with a higher score representing a better learning ability.

**Method A5: Definition of educational and occupational level of parents**

***Educational level of parents***

The education level is the average of the paternal and maternal educational level, and calculated based upon the 3-point scale subdivisions as made by the Algemene Directie Statistiek (Belgium; statbel.fgov.be/nl/) and the Centraal Bureau voor de Statistiek (The Netherlands; statline.cbs.nl): Low (=1), middle (=2) and high (=3) educational level.

***Occupational level of parents***

The occupation level is the average of the paternal and maternal occupation level, which is calculated based upon the International Isco System 4-point scale for professions.^[18](#_ENREF_18)^ In case one of the parents filled in two jobs in the questionnaire, the highest Isco code level was used. In case “unemployed”, “disabled”, “student”, or “housewife/houseman” was filled in, an Isco code level of 1 was given to that parent. When the parents described their profession as “employee”, “worker”, “liberal profession”, or “retired”, they were given an Isco code level of 2.

**Figure A1: CONSORT diagram of study participants**


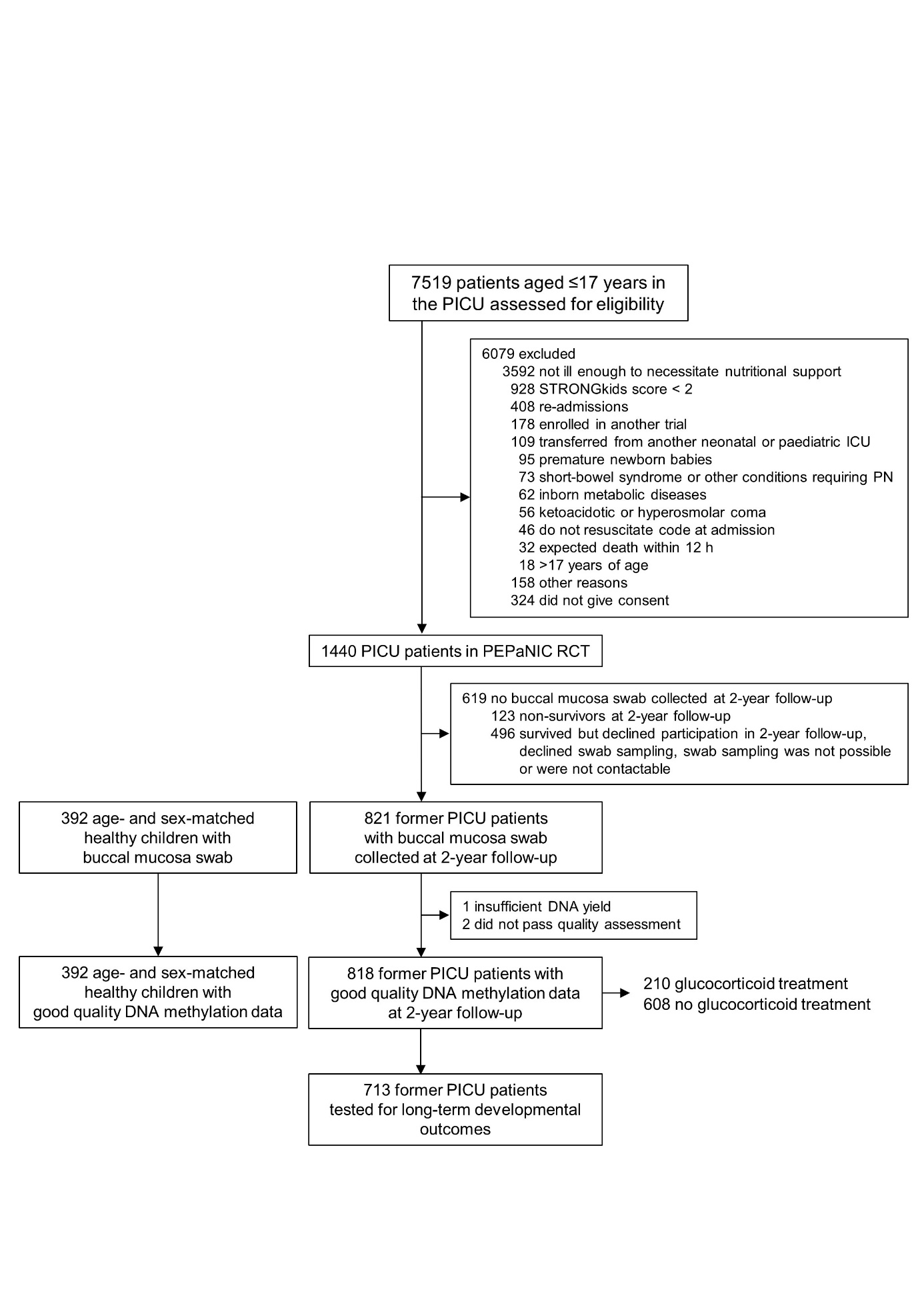


Abbreviations: PICU, paediatric intensive care unit; PEPaNIC, Paediatric Early versus Late Parenteral Nutrition in Intensive Care Unit; RCT, randomised controlled trial.

**Figure A2: Univariate boxplots of the methylation status of differentially methylated positions in former PICU patients as compared with matched healthy children**


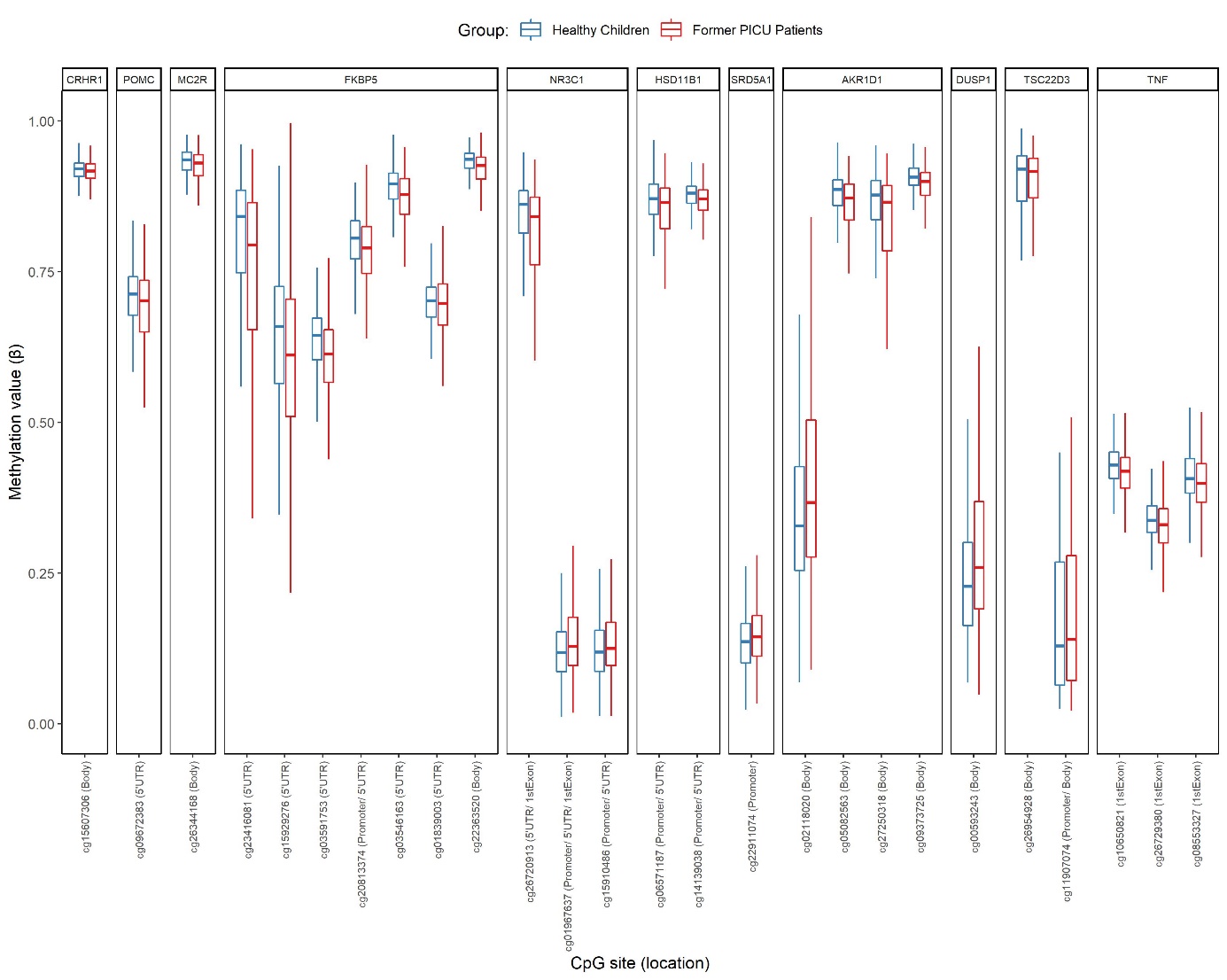


The boxplots show a univariate presentation of the methylation status (β-value) of the CpG sites that were differentially methylated between former PICU patients (n=818, red) and matched healthy children (n=392, blue). The CpG sites are grouped per gene (top x-axis) and positioned based on their location within the gene (bottom x-axis). The central lines of the boxplots depict the medians, the boxes the interquartile ranges, and the whiskers are drawn to the furthest point within 1.5 times the interquartile range from the box.

Abbreviations: PICU, paediatric intensive care unit; UTR: untranslated region.

**Figure A3: Univariate boxplots of the methylation status of the CpG sites within the regions identified as differentially methylated between former PICU patients and matched healthy children**


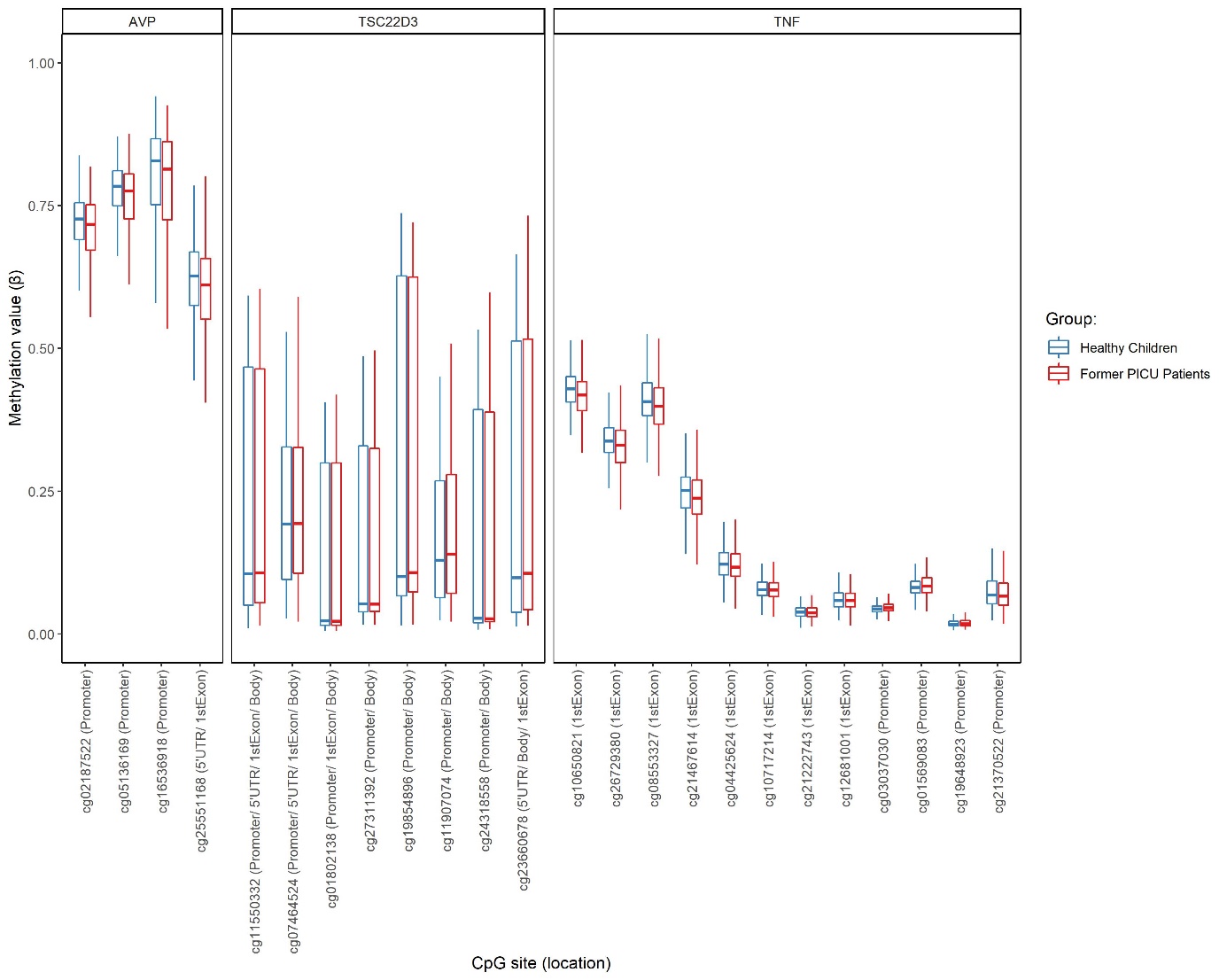


The boxplots show a univariate presentation of the methylation status (β-value) of the CpG sites located within the DNA regions identified as differentially methylated between former PICU patients (n=818, red) and matched healthy children (n=392, blue). The CpG sites are positioned based on their location within the gene (bottom x-axis). Differentially methylated regions (DMRs) were identified with the DMRcate package (Supplementary Method S3). Fisher’s p-values are 0.0077 for the DMR within *AVP*, 0.0072 for the DMR within *TSC22D3*, and 0.0041 for the DMR within *TNF*. The central lines of the boxplots depict the medians, the boxes the interquartile ranges, and the whiskers are drawn to the furthest point within 1.5 times the interquartile range from the box.

Abbreviations: DMR, differentially methylated region; PICU, paediatric intensive care unit.

**Table A1: DMP analysis – Former PICU patients vs Healthy children**

| **Gene name** | **CpG** | **Chr** | **Position ^a^** | **Gene section ^b, c^** | **Log Fold Change**  **[Confidence Interval] ^d^** | **p-value ^e^** | **FANTOM4**  **Enhancer location ^f^** | **FANTOM5**  **Enhancer location ^g^** | **ENCODE Regulatory Feature location ^h^** | **DMR ^i^** |
| --- | --- | --- | --- | --- | --- | --- | --- | --- | --- | --- |
| CRH | cg03405789 | chr8 | 67088895 | 3'UTR | 0.001 [-0.050 – 0.051] | 0.98 |  |  | 8:67088727-67090297 |  |
|  | cg21240762 | chr8 | 67089388 | Body | 0.022 [-0.006 – 0.050] | 0.33 |  |  | 8:67088727-67090297 |  |
|  | cg23027580 | chr8 | 67089513 | Body | -0.002 [-0.041 – 0.036] | 0.94 |  |  | 8:67088727-67090297 |  |
|  | cg15971888 | chr8 | 67089599 | Body | 0.009 [-0.019 – 0.036] | 0.75 |  |  | 8:67088727-67090297 |  |
|  | cg00603617 | chr8 | 67089831 | 5'UTR | -0.007 [-0.050 – 0.035] | 0.86 |  |  | 8:67088727-67090297 |  |
|  | cg21878188 | chr8 | 67089887 | 5'UTR | 0.006 [-0.029 – 0.041] | 0.85 |  |  | 8:67088727-67090297 |  |
|  | cg20329958 | chr8 | 67090250 | 5'UTR | 0.026 [-0.016 – 0.068] | 0.46 |  |  | 8:67088727-67090297 |  |
|  | cg17305181 | chr8 | 67090581 | 5'UTR/1stExon | 0.035 [-0.060 – 0.129] | 0.70 |  |  |  |  |
|  | cg18640030 | chr8 | 67090752 | Promoter | -0.043 [-0.110 – 0.024] | 0.43 |  |  |  |  |
|  | cg08215831 | chr8 | 67090776 | Promoter | -0.035 [-0.074 – 0.004] | 0.27 |  |  |  |  |
|  | cg19035496 | chr8 | 67090792 | Promoter | -0.025 [-0.062 – 0.013] | 0.42 |  |  |  |  |
|  | cg23409074 | chr8 | 67090798 | Promoter | -0.044 [-0.102 – 0.013] | 0.34 |  |  |  |  |
|  | cg11699476 | chr8 | 67090909 | Promoter | 0.004 [-0.033 – 0.041] | 0.91 |  |  |  |  |
|  | cg04227637 | chr8 | 67090922 | Promoter | -0.008 [-0.060 – 0.044] | 0.86 |  |  |  |  |
|  | cg00269606 | chr8 | 67090928 | Promoter | -0.026 [-0.058 – 0.006] | 0.33 |  |  |  |  |
|  | cg17578833 | chr8 | 67091058 | Promoter | -0.035 [-0.101 – 0.031] | 0.54 |  |  |  |  |
|  | cg19696975 | chr8 | 67091449 | Promoter | -0.020 [-0.066 – 0.025] | 0.61 |  |  |  |  |
|  | cg02834846 | chr8 | 67091467 | Promoter | -0.020 [-0.052 – 0.011] | 0.43 |  |  |  |  |
|  | cg16664570 | chr8 | 67091580 | Promoter | -0.004 [-0.047 – 0.039] | 0.92 |  |  |  |  |
|  | cg01972826 | chr8 | 67091910 | Promoter | 0.019 [-0.044 – 0.081] | 0.77 |  |  |  |  |
|  | cg23990470 | chr8 | 67092082 | Promoter | -0.033 [-0.075 – 0.010] | 0.34 |  |  |  |  |
| CRHR1 | cg08929103 | chr17 | 43860355 | Promoter | -0.017 [-0.053 – 0.019] | 0.58 |  |  |  |  |
|  | cg22046703 | chr17 | 43860472 | Promoter | -0.019 [-0.057 – 0.018] | 0.55 |  |  |  |  |
|  | cg12577105 | chr17 | 43860685 | Promoter | 0.022 [-0.018 – 0.062] | 0.52 |  |  | 17:43860547-43860875 |  |
|  | cg18757974 | chr17 | 43860691 | Promoter | -0.016 [-0.063 – 0.030] | 0.72 |  |  | 17:43860547-43860875 |  |
|  | cg08473090 | chr17 | 43861104 | Promoter | 0.018 [-0.010 – 0.046] | 0.44 |  |  |  |  |
|  | cg11338426 | chr17 | 43861668 | 5'UTR/1stExon | 0.026 [-0.024 – 0.076] | 0.55 | high-CpG:41217391-41217448 |  |  |  |
|  | cg13521908 | chr17 | 43861682 | 5'UTR/1stExon | 0.031 [-0.003 – 0.066] | 0.25 | high-CpG:41217391-41217448 |  |  |  |
|  | cg04856689 | chr17 | 43862032 | Body | 0.006 [-0.025 – 0.037] | 0.85 |  |  |  |  |
|  | cg24353392 | chr17 | 43862247 | Body | 0.024 [-0.021 – 0.070] | 0.53 |  |  |  |  |
|  | cg11731737 | chr17 | 43862617 | Body | 0.027 [0.006 – 0.047] | 0.083 |  |  |  |  |
|  | cg27551605 | chr17 | 43862910 | Body | 0.009 [-0.033 – 0.052] | 0.81 |  |  |  |  |
|  | cg07778819 | chr17 | 43862927 | Body | 0.004 [-0.044 – 0.051] | 0.92 |  |  |  |  |
|  | cg24394631 | chr17 | 43863000 | Body | 0.018 [-0.032 – 0.069] | 0.71 |  |  |  |  |
|  | cg24063856 | chr17 | 43863303 | Body | 0.034 [-0.017 – 0.085] | 0.42 |  |  |  |  |
|  | cg13947929 | chr17 | 43863356 | Body | 0.100 [0.030 – 0.171] | 0.069 |  |  |  |  |
|  | cg11760414 | chr17 | 43863398 | 5'UTR/Body | 0.071 [-0.006 – 0.148] | 0.25 |  | chr17:43863369-43863369 |  |  |
|  | cg23420656 | chr17 | 43865031 | 5'UTR/Body | -0.021 [-0.065 – 0.022] | 0.56 |  |  |  |  |
|  | cg27410679 | chr17 | 43866278 | Body | -0.049 [-0.087 – -0.011] | 0.090 |  |  |  |  |
|  | cg14297797 | chr17 | 43867801 | 5'UTR/Body | 0.230 [0.067 – 0.393] | 0.069 |  |  |  |  |
|  | cg09422970 | chr17 | 43870537 | 5'UTR/Body | 0.007 [-0.036 – 0.050] | 0.86 |  |  |  |  |
|  | cg15117716 | chr17 | 43871537 | 5'UTR/Body | 0.251 [0.083 – 0.419] | 0.052 |  |  |  |  |
|  | cg16642545 | chr17 | 43878769 | Body | -0.029 [-0.067 – 0.008] | 0.34 |  |  |  |  |
|  | cg10106856 | chr17 | 43880210 | 5'UTR/Body | -0.006 [-0.040 – 0.029] | 0.86 |  |  |  |  |
|  | cg02810898 | chr17 | 43881561 | 5'UTR/Body | 0.017 [-0.021 – 0.055] | 0.61 |  |  |  |  |
|  | cg18534039 | chr17 | 43883072 | Body | -0.005 [-0.048 – 0.039] | 0.91 |  |  |  |  |
|  | cg15607306 | chr17 | 43883843 | Body | -0.066 [-0.108 – -0.023] | 0.050 |  |  |  |  |
|  | cg00022871 | chr17 | 43884358 | Body | -0.003 [-0.042 – 0.037] | 0.94 |  |  |  |  |
|  | cg07657976 | chr17 | 43887205 | 5'UTR/Body | 0.033 [-0.020 – 0.087] | 0.45 |  |  |  |  |
|  | cg03323388 | chr17 | 43888776 | 5'UTR/Body | 0.018 [-0.020 – 0.056] | 0.57 |  |  |  |  |
|  | cg27503360 | chr17 | 43890749 | Body | -0.014 [-0.042 – 0.013] | 0.55 |  |  |  |  |
|  | cg10256584 | chr17 | 43892357 | 5'UTR/Body | -0.011 [-0.053 – 0.031] | 0.79 |  |  |  |  |
|  | cg09251165 | chr17 | 43894089 | 5'UTR/Body | -0.028 [-0.065 – 0.009] | 0.34 |  |  |  |  |
|  | cg24738082 | chr17 | 43898761 | 5'UTR/Body | -0.010 [-0.058 – 0.039] | 0.84 |  |  |  |  |
|  | cg17311440 | chr17 | 43900684 | 5'UTR/Body | -0.064 [-0.129 – 0.001] | 0.22 |  |  |  |  |
|  | cg08043197 | chr17 | 43901722 | 5'UTR/Body | -0.039 [-0.070 – -0.007] | 0.11 |  |  |  |  |
|  | cg03066966 | chr17 | 43902069 | 5'UTR/Body | -0.002 [-0.047 – 0.043] | 0.95 |  |  |  |  |
|  | cg11524343 | chr17 | 43905642 | 5'UTR/Body | -0.097 [-0.173 – -0.020] | 0.096 |  |  |  |  |
|  | cg00025823 | chr17 | 43909151 | Body | 0.012 [-0.025 – 0.049] | 0.75 |  |  | 17:43908792-43910231 |  |
|  | cg02122296 | chr17 | 43909656 | Body | 0.003 [-0.029 – 0.036] | 0.91 |  |  | 17:43908792-43910231 |  |
|  | cg26656751 | chr17 | 43910226 | Body | 0.016 [-0.041 – 0.073] | 0.78 |  |  | 17:43908792-43910231 |  |
|  | cg16830379 | chr17 | 43912434 | 3'UTR | -0.026 [-0.066 – 0.014] | 0.42 |  |  |  |  |
| AVP | cg14065127 | chr20 | 3063238 | 3'UTR | -0.026 [-0.081 – 0.030] | 0.59 |  |  |  |  |
|  | cg25673357 | chr20 | 3063586 | Body | 0.002 [-0.041 – 0.044] | 0.96 |  |  |  |  |
|  | cg03279206 | chr20 | 3064001 | Body | -0.056 [-0.096 – -0.017] | 0.065 |  |  |  |  |
|  | cg04360210 | chr20 | 3064015 | Body | -0.033 [-0.074 – 0.007] | 0.32 |  |  |  |  |
|  | cg25551168 | chr20 | 3065343 | 5'UTR/1stExon | -0.067 [-0.120 – -0.014] | 0.098 |  |  |  | DMR_3 |
|  | cg16536918 | chr20 | 3065403 | Promoter | -0.113 [-0.202 – -0.023] | 0.097 |  |  |  | DMR_3 |
|  | cg05136169 | chr20 | 3065473 | Promoter | -0.068 [-0.116 – -0.021] | 0.062 |  |  |  | DMR_3 |
|  | cg02187522 | chr20 | 3065488 | Promoter | -0.063 [-0.105 – -0.021] | 0.052 |  |  |  | DMR_3 |
|  | cg04632887 | chr20 | 3065559 | Promoter | -0.087 [-0.153 – -0.020] | 0.084 |  |  |  |  |
|  | cg23169111 | chr20 | 3065582 | Promoter | -0.035 [-0.066 – -0.005] | 0.14 |  |  |  |  |
|  | cg15313891 | chr20 | 3065584 | Promoter | -0.042 [-0.083 – -0.001] | 0.20 |  |  |  |  |
|  | cg24025566 | chr20 | 3065698 | Promoter | -0.018 [-0.055 – 0.020] | 0.59 |  |  |  |  |
|  | cg23035419 | chr20 | 3066014 | Promoter | -0.025 [-0.058 – 0.008] | 0.36 |  |  |  |  |
|  | cg15189567 | chr20 | 3066643 | Promoter | -0.027 [-0.066 – 0.012] | 0.40 |  |  |  |  |
| AVPR1b | cg18992688 | chr1 | 206223241 | Promoter | -0.068 [-0.119 – -0.017] | 0.082 |  |  |  |  |
|  | cg27169129 | chr1 | 206223453 | Promoter | -0.072 [-0.134 – -0.010] | 0.14 |  |  | 1:206223415-206223605 |  |
|  | cg10818339 | chr1 | 206223499 | Promoter | -0.019 [-0.082 – 0.044] | 0.76 |  |  | 1:206223415-206223605 |  |
|  | cg17587023 | chr1 | 206223592 | Promoter | 0.028 [-0.008 – 0.065] | 0.34 |  |  | 1:206223415-206223605 |  |
|  | cg00761787 | chr1 | 206223701 | Promoter | -0.012 [-0.044 – 0.020] | 0.70 |  |  | 1:206223680-206224049 |  |
|  | cg26795730 | chr1 | 206223719 | Promoter | 0.015 [-0.040 – 0.071] | 0.79 |  |  | 1:206223680-206224049 |  |
|  | cg16368479 | chr1 | 206223808 | Promoter | 0.042 [-0.008 – 0.092] | 0.31 |  |  | 1:206223680-206224049 |  |
|  | cg17940251 | chr1 | 206224005 | Promoter | 0.018 [-0.005 – 0.041] | 0.33 |  |  | 1:206223680-206224049 |  |
|  | cg04633513 | chr1 | 206224027 | Promoter | 0.005 [-0.024 – 0.034] | 0.86 |  |  | 1:206223680-206224049 |  |
|  | cg08709672 | chr1 | 206224334 | 5'UTR/1stExon | -0.016 [-0.040 – 0.007] | 0.41 |  |  |  |  |
|  | cg11215296 | chr1 | 206224554 | 1stExon | -0.017 [-0.055 – 0.022] | 0.62 |  |  |  |  |
|  | cg15677434 | chr1 | 206226009 | Body | -0.059 [-0.110 – -0.008] | 0.14 |  |  |  |  |
|  | cg20295214 | chr1 | 206226794 | Body | -0.026 [-0.058 – 0.005] | 0.30 |  |  |  |  |
|  | cg26596278 | chr1 | 206227162 | Body | 0.229 [0.064 – 0.395] | 0.072 |  |  |  |  |
|  | cg09631259 | chr1 | 206229625 | Body | 0.186 [0.041 – 0.332] | 0.090 |  |  |  |  |
| POMC | cg23809645 | chr2 | 25383851 | 3'UTR | -0.014 [-0.068 – 0.041] | 0.79 |  |  | 2:25383847-25384998 |  |
|  | cg10045137 | chr2 | 25383940 | 3'UTR | 0.027 [-0.052 – 0.106] | 0.72 |  |  | 2:25383847-25384998 |  |
|  | cg02716646 | chr2 | 25384293 | Body | 0.010 [-0.033 – 0.054] | 0.79 |  |  | 2:25383847-25384998 |  |
|  | cg06846259 | chr2 | 25384654 | Body | 0.038 [-0.019 – 0.095] | 0.42 |  |  | 2:25383847-25384998 |  |
|  | cg20807790 | chr2 | 25384762 | Body | 0.019 [-0.035 – 0.072] | 0.72 |  |  | 2:25383847-25384998 |  |
|  | cg02757179 | chr2 | 25384809 | Body | 0.016 [-0.057 – 0.089] | 0.81 |  |  | 2:25383847-25384998 |  |
|  | cg14170547 | chr2 | 25387636 | Body | -0.036 [-0.076 – 0.004] | 0.27 |  |  |  |  |
|  | cg14357535 | chr2 | 25389040 | 5'UTR | -0.011 [-0.055 – 0.033] | 0.79 |  |  | 2:25388733-25389382 |  |
|  | cg11894631 | chr2 | 25389989 | 5'UTR | 0.003 [-0.034 – 0.040] | 0.93 |  |  |  |  |
|  | cg09527270 | chr2 | 25390385 | 5'UTR | -0.126 [-0.239 – -0.014] | 0.15 |  |  |  |  |
|  | cg03560973 | chr2 | 25390400 | 5'UTR | -0.109 [-0.190 – -0.029] | 0.078 |  |  |  |  |
|  | cg02079741 | chr2 | 25390424 | 5'UTR | -0.064 [-0.112 – -0.015] | 0.083 |  |  |  |  |
|  | cg09672383 | chr2 | 25390540 | 5'UTR | -0.082 [-0.135 – -0.030] | 0.050 |  |  |  |  |
|  | cg24718866 | chr2 | 25391274 | 5'UTR | 0.005 [-0.034 – 0.044] | 0.90 |  |  | 2:25390959-25392100 |  |
|  | cg00293936 | chr2 | 25391505 | 5'UTR/1stExon | -0.017 [-0.058 – 0.024] | 0.65 |  |  | 2:25390959-25392100 |  |
|  | cg06904565 | chr2 | 25391522 | 5'UTR/1stExon | 0.003 [-0.049 – 0.055] | 0.94 |  |  | 2:25390959-25392100 |  |
|  | cg13025668 | chr2 | 25391656 | Promoter | 0.008 [-0.021 – 0.038] | 0.78 |  |  | 2:25390959-25392100 |  |
|  | cg20387815 | chr2 | 25391666 | Promoter | 0.059 [-0.027 – 0.144] | 0.41 |  |  | 2:25390959-25392100 |  |
|  | cg01926269 | chr2 | 25391670 | Promoter | 0.028 [-0.024 – 0.080] | 0.53 |  |  | 2:25390959-25392100 |  |
|  | cg00674304 | chr2 | 25391700 | Promoter | 0.002 [-0.046 – 0.050] | 0.96 |  |  | 2:25390959-25392100 |  |
|  | cg24425171 | chr2 | 25391711 | Promoter | 0.042 [-0.008 – 0.091] | 0.30 |  |  | 2:25390959-25392100 |  |
|  | cg22900229 | chr2 | 25391773 | Promoter | 0.072 [0.018 – 0.125] | 0.081 |  |  | 2:25390959-25392100 |  |
|  | cg16302441 | chr2 | 25391830 | Promoter | 0.076 [-0.026 – 0.179] | 0.36 |  |  | 2:25390959-25392100 |  |
|  | cg08030082 | chr2 | 25391839 | Promoter | 0.081 [-0.002 – 0.164] | 0.22 |  |  | 2:25390959-25392100 |  |
|  | cg09916783 | chr2 | 25391911 | Promoter | 0.018 [-0.036 – 0.072] | 0.73 |  |  | 2:25390959-25392100 |  |
|  | cg19241807 | chr2 | 25392556 | Promoter | -0.001 [-0.038 – 0.037] | 0.98 |  |  |  |  |
|  | cg15715889 | chr2 | 25392941 | Promoter | -0.066 [-0.110 – -0.023] | 0.052 |  |  |  |  |
| MC2R | cg25924472 | chr18 | 13884152 | 3'UTR | -0.034 [-0.068 – 0.001] | 0.22 |  |  |  |  |
|  | cg26344168 | chr18 | 13884969 | Body | -0.070 [-0.113 – -0.027] | 0.049 |  |  |  |  |
|  | cg22624572 | chr18 | 13886087 | 5'UTR | 0.031 [-0.011 – 0.073] | 0.36 |  |  |  |  |
|  | cg08578419 | chr18 | 13886969 | 5'UTR | -0.034 [-0.060 – -0.008] | 0.084 |  |  |  |  |
|  | cg04228045 | chr18 | 13889915 | 5'UTR | 0.127 [0.011 – 0.242] | 0.16 |  |  |  |  |
|  | cg13187423 | chr18 | 13890540 | 5'UTR | -0.010 [-0.057 – 0.037] | 0.81 |  | chr18:13890423-13890423 |  |  |
|  | cg16500026 | chr18 | 13892132 | 5'UTR | -0.017 [-0.078 – 0.044] | 0.78 |  |  |  |  |
|  | cg00527908 | chr18 | 13893625 | 5'UTR | -0.029 [-0.064 – 0.006] | 0.31 |  | chr18:13893492-13893492 | 18:13893336-13893707 |  |
|  | cg14829190 | chr18 | 13893806 | 5'UTR | 0.006 [-0.039 – 0.052] | 0.88 |  |  |  |  |
|  | cg12343754 | chr18 | 13895861 | 5'UTR | -0.001 [-0.051 – 0.049] | 0.98 |  |  |  |  |
|  | cg17828058 | chr18 | 13909311 | 5'UTR | -0.026 [-0.075 – 0.023] | 0.54 |  |  |  |  |
|  | cg11564670 | chr18 | 13915006 | 5'UTR | -0.038 [-0.078 – 0.002] | 0.24 |  |  |  |  |
|  | cg24922596 | chr18 | 13915573 | Promoter | -0.019 [-0.051 – 0.012] | 0.46 |  |  |  |  |
|  | cg05667256 | chr18 | 13915583 | Promoter | -0.005 [-0.036 – 0.026] | 0.86 |  |  |  |  |
|  | cg17444561 | chr18 | 13915600 | Promoter/5'UTR | -0.051 [-0.100 – -0.002] | 0.19 |  |  |  |  |
|  | cg20494779 | chr18 | 13916113 | Promoter | 0.151 [0.025 – 0.278] | 0.12 |  |  |  |  |
|  | cg03664443 | chr18 | 13916330 | Promoter | 0.025 [-0.048 – 0.098] | 0.72 |  |  |  |  |
|  | cg00335286 | chr18 | 13916355 | Promoter | 0.153 [0.035 – 0.270] | 0.087 |  |  |  |  |
|  | cg22364075 | chr18 | 13916462 | Promoter | 0.057 [-0.017 – 0.131] | 0.34 |  |  |  |  |
|  | cg22434252 | chr18 | 13916535 | Promoter | 0.023 [-0.040 – 0.086] | 0.70 |  |  |  |  |
| FKBP5 | cg02665568 | chr6 | 35544468 | Body | 0.016 [-0.028 – 0.060] | 0.71 |  |  |  |  |
|  | cg16912838 | chr6 | 35551624 | Body/3'UTR | 0.011 [-0.051 – 0.073] | 0.85 |  |  | 6:35551514-35552078 |  |
|  | cg06087101 | chr6 | 35551932 | Body/3'UTR | 0.086 [-0.007 – 0.178] | 0.24 |  |  | 6:35551514-35552078 |  |
|  | cg22363520 | chr6 | 35558488 | Body | -0.253 [-0.338 – -0.167] | <0.0001 |  |  |  |  |
|  | cg10300814 | chr6 | 35565116 | Body | -0.067 [-0.136 – 0.002] | 0.22 | low-CpG:35673085-35673096 |  |  |  |
|  | cg07633853 | chr6 | 35569471 | Body | 0.024 [-0.033 – 0.081] | 0.64 |  | chr6:35569141-35569141 | 6:35569463-35570060 |  |
|  | cg14284211 | chr6 | 35570224 | Body | 0.039 [0.004 – 0.073] | 0.15 |  |  |  |  |
|  | cg13344434 | chr6 | 35570573 | Body | 0.020 [-0.024 – 0.063] | 0.61 |  | chr6:35570271-35570271 |  |  |
|  | cg16005389 | chr6 | 35592694 | Body | -0.006 [-0.051 – 0.039] | 0.89 |  |  |  |  |
|  | cg16052510 | chr6 | 35603143 | Body | -0.017 [-0.084 – 0.049] | 0.79 |  |  |  |  |
|  | cg04791658 | chr6 | 35611554 | 5'UTR | -0.009 [-0.040 – 0.023] | 0.79 |  |  |  |  |
|  | cg09268536 | chr6 | 35611576 | 5'UTR | -0.007 [-0.053 – 0.038] | 0.86 |  |  |  |  |
|  | cg04137760 | chr6 | 35611611 | 5'UTR | -0.026 [-0.066 – 0.013] | 0.42 |  |  |  |  |
|  | cg08586216 | chr6 | 35612351 | 5'UTR | 0.006 [-0.019 – 0.032] | 0.79 |  |  |  |  |
|  | cg05039098 | chr6 | 35618763 | 5'UTR | 0.016 [-0.041 – 0.074] | 0.78 |  |  |  |  |
|  | cg07696519 | chr6 | 35619165 | 5'UTR | -0.036 [-0.137 – 0.066] | 0.72 |  |  |  |  |
|  | cg26495008 | chr6 | 35619239 | 5'UTR | -0.020 [-0.058 – 0.017] | 0.53 |  |  |  |  |
|  | cg01839003 | chr6 | 35631009 | 5'UTR | -0.077 [-0.125 – -0.029] | 0.050 |  |  |  |  |
|  | cg07061368 | chr6 | 35631736 | 5'UTR | -0.007 [-0.053 – 0.039] | 0.86 |  |  |  |  |
|  | cg21789597 | chr6 | 35633557 | 5'UTR | -0.003 [-0.063 – 0.057] | 0.95 |  |  |  |  |
|  | cg19014730 | chr6 | 35635985 | 5'UTR | -0.021 [-0.065 – 0.024] | 0.60 |  |  |  |  |
|  | cg06409316 | chr6 | 35642470 | 5'UTR | 0.155 [0.040 – 0.269] | 0.078 |  |  |  |  |
|  | cg17085721 | chr6 | 35645341 | 5'UTR | 0.011 [-0.025 – 0.046] | 0.76 |  |  |  |  |
|  | cg14642437 | chr6 | 35652521 | 5'UTR | -0.038 [-0.071 – -0.005] | 0.14 |  |  | 6:35652253-35653196 |  |
|  | cg03546163 | chr6 | 35654363 | 5'UTR | -0.178 [-0.248 – -0.108] | 0.00012 |  |  |  |  |
|  | cg00862770 | chr6 | 35655764 | 5'UTR | 0.021 [-0.009 – 0.050] | 0.40 |  |  | 6:35655414-35657286 |  |
|  | cg00140191 | chr6 | 35656242 | 5'UTR | -0.014 [-0.069 – 0.041] | 0.79 |  |  | 6:35655414-35657286 |  |
|  | cg10913456 | chr6 | 35656590 | 5'UTR/1stExon | 0.011 [-0.028 – 0.050] | 0.79 | high-CpG:35764243-35764755 |  | 6:35655414-35657286 |  |
|  | cg16012111 | chr6 | 35656758 | Promoter/5'UTR | -0.012 [-0.054 – 0.030] | 0.78 | high-CpG:35764243-35764755 |  | 6:35655414-35657286 |  |
|  | cg07843056 | chr6 | 35656848 | Promoter/5'UTR | 0.022 [-0.001 – 0.046] | 0.23 |  |  | 6:35655414-35657286 |  |
|  | cg01294490 | chr6 | 35656906 | Promoter/5'UTR | 0.000 [-0.038 – 0.039] | 0.99 |  |  | 6:35655414-35657286 |  |
|  | cg03245912 | chr6 | 35657040 | Promoter/5'UTR | -0.039 [-0.065 – -0.013] | 0.052 |  |  | 6:35655414-35657286 |  |
|  | cg20813374 | chr6 | 35657180 | Promoter/5'UTR | -0.126 [-0.183 – -0.070] | 0.0020 |  |  | 6:35655414-35657286 |  |
|  | cg00130530 | chr6 | 35657202 | Promoter/5'UTR | -0.060 [-0.113 – -0.007] | 0.15 |  |  | 6:35655414-35657286 |  |
|  | cg08636224 | chr6 | 35657921 | Promoter/5'UTR | -0.012 [-0.046 – 0.021] | 0.70 |  |  |  |  |
|  | cg03591753 | chr6 | 35659141 | 5'UTR | -0.132 [-0.176 – -0.088] | <0.0001 |  |  |  |  |
|  | cg08642543 | chr6 | 35662800 | 5'UTR | 0.008 [-0.027 – 0.043] | 0.81 |  |  |  |  |
|  | cg03098337 | chr6 | 35677981 | 5'UTR | -0.017 [-0.049 – 0.015] | 0.53 |  |  |  |  |
|  | cg24295963 | chr6 | 35681420 | 5'UTR | -0.004 [-0.041 – 0.033] | 0.91 |  | chr6:35681097-35681097 |  |  |
|  | cg14339974 | chr6 | 35687310 | 5'UTR | -0.116 [-0.197 – -0.036] | 0.063 |  |  |  |  |
|  | cg15929276 | chr6 | 35687457 | 5'UTR | -0.252 [-0.397 – -0.107] | 0.038 |  |  | 6:35687313-35687891 |  |
|  | cg23416081 | chr6 | 35693573 | 5'UTR | -0.292 [-0.432 – -0.152] | 0.0053 |  | chr6:35693475-35693475 |  |  |
|  | cg06937024 | chr6 | 35695489 | 5'UTR | 0.027 [0.001 – 0.052] | 0.19 |  |  | 6:35695296-35697254 |  |
|  | cg11845071 | chr6 | 35695859 | 5'UTR | 0.037 [0.000 – 0.075] | 0.22 |  |  | 6:35695296-35697254 |  |
|  | cg00610228 | chr6 | 35695934 | 5'UTR | -0.011 [-0.054 – 0.032] | 0.79 |  |  | 6:35695296-35697254 |  |
|  | cg07485685 | chr6 | 35696061 | 5'UTR | 0.036 [-0.021 – 0.093] | 0.44 |  |  | 6:35695296-35697254 |  |
|  | cg17030679 | chr6 | 35696300 | 5'UTR/1stExon | 0.036 [-0.001 – 0.072] | 0.22 |  |  | 6:35695296-35697254 |  |
|  | cg25563198 | chr6 | 35696726 | Promoter | 0.030 [-0.037 – 0.097] | 0.61 |  |  | 6:35695296-35697254 |  |
|  | cg25114611 | chr6 | 35696870 | Promoter | 0.067 [-0.001 – 0.135] | 0.21 |  |  | 6:35695296-35697254 |  |
|  | cg19226017 | chr6 | 35697185 | Promoter | 0.067 [-0.020 – 0.153] | 0.34 |  |  | 6:35695296-35697254 |  |
|  | cg21517946 | chr6 | 35697810 | Promoter | 0.214 [-0.128 – 0.556] | 0.45 |  |  |  |  |
| NR3C1 | cg23273257 | chr5 | 142658828 | 3'UTR | -0.007 [-0.035 – 0.021] | 0.79 |  |  |  |  |
|  | cg26081259 | chr5 | 142659682 | Body/3'UTR | -0.008 [-0.035 – 0.019] | 0.77 |  |  |  |  |
|  | cg13514002 | chr5 | 142677292 | Body | -0.010 [-0.069 – 0.049] | 0.86 |  |  |  |  |
|  | cg24801588 | chr5 | 142689858 | Body | -0.008 [-0.052 – 0.036] | 0.86 |  |  |  |  |
|  | cg00407401 | chr5 | 142690959 | Body | -0.004 [-0.052 – 0.043] | 0.92 |  | chr5:142690864-142690864 |  |  |
|  | cg19457823 | chr5 | 142692961 | Body | 0.048 [-0.012 – 0.109] | 0.33 |  |  |  |  |
|  | cg12741214 | chr5 | 142695619 | Body | -0.029 [-0.061 – 0.003] | 0.26 |  |  |  |  |
|  | cg04457787 | chr5 | 142695636 | Body | -0.012 [-0.053 – 0.029] | 0.77 |  |  |  |  |
|  | cg20728768 | chr5 | 142696594 | Body | 0.014 [-0.037 – 0.064] | 0.79 |  |  |  |  |
|  | cg25708981 | chr5 | 142697868 | Body | 0.015 [-0.068 – 0.097] | 0.85 |  | chr5:142697698-142697698 |  |  |
|  | cg19645279 | chr5 | 142702733 | Body | -0.003 [-0.034 – 0.027] | 0.91 |  |  |  |  |
|  | cg19176661 | chr5 | 142718549 | Body | -0.030 [-0.062 – 0.001] | 0.23 |  |  |  |  |
|  | cg07715663 | chr5 | 142721796 | Body | 0.006 [-0.067 – 0.080] | 0.92 |  |  |  |  |
|  | cg24052866 | chr5 | 142727470 | Body | 0.068 [0.003 – 0.134] | 0.19 |  |  |  |  |
|  | cg22233604 | chr5 | 142729377 | Body | -0.037 [-0.074 – -0.001] | 0.20 |  |  |  |  |
|  | cg03857453 | chr5 | 142729913 | Body | -0.071 [-0.131 – -0.011] | 0.12 |  |  |  |  |
|  | cg15115787 | chr5 | 142730701 | Body | 0.021 [-0.018 – 0.059] | 0.54 |  |  |  |  |
|  | cg12888360 | chr5 | 142732502 | Body | 0.010 [-0.045 – 0.065] | 0.85 |  |  |  |  |
|  | cg08695103 | chr5 | 142733619 | Body | 0.052 [0.002 – 0.102] | 0.19 |  |  |  |  |
|  | cg14621978 | chr5 | 142735238 | Body | 0.177 [0.032 – 0.322] | 0.11 |  |  |  |  |
|  | cg18484679 | chr5 | 142740314 | Body | -0.004 [-0.044 – 0.037] | 0.92 |  |  |  |  |
|  | cg16586394 | chr5 | 142757011 | Body | -0.023 [-0.055 – 0.009] | 0.39 |  |  |  |  |
|  | cg25535999 | chr5 | 142757312 | Body | -0.020 [-0.048 – 0.008] | 0.39 |  |  |  |  |
|  | cg03746860 | chr5 | 142759375 | Body | 0.015 [-0.027 – 0.057] | 0.72 |  |  |  |  |
|  | cg20598211 | chr5 | 142762454 | Body | 0.060 [-0.010 – 0.130] | 0.28 |  |  |  |  |
|  | cg05483455 | chr5 | 142762613 | Body | -0.007 [-0.065 – 0.051] | 0.90 |  |  |  |  |
|  | cg16594263 | chr5 | 142768048 | Body | -0.017 [-0.046 – 0.013] | 0.51 |  |  |  |  |
|  | cg16535116 | chr5 | 142769612 | Body | -0.030 [-0.079 – 0.018] | 0.45 |  |  |  |  |
|  | cg05900547 | chr5 | 142769791 | Body | -0.024 [-0.071 – 0.024] | 0.56 |  |  |  |  |
|  | cg19432243 | chr5 | 142770757 | Body | 0.050 [-0.050 – 0.150] | 0.56 |  |  |  |  |
|  | cg19820298 | chr5 | 142770782 | Body | -0.057 [-0.154 – 0.040] | 0.49 |  |  |  |  |
|  | cg27107893 | chr5 | 142776274 | Body | 0.043 [-0.039 – 0.125] | 0.55 |  |  |  |  |
|  | cg06613263 | chr5 | 142779552 | Body | -0.032 [-0.105 – 0.042] | 0.63 |  |  |  |  |
|  | cg17342132 | chr5 | 142780254 | Body | -0.098 [-0.216 – 0.021] | 0.32 |  |  | 5:142780252-142780340 |  |
|  | cg07742588 | chr5 | 142780439 | 5'UTR | 0.010 [-0.070 – 0.090] | 0.89 |  |  |  |  |
|  | cg00294552 | chr5 | 142780486 | 5'UTR | -0.038 [-0.115 – 0.039] | 0.56 |  |  |  |  |
|  | cg16219186 | chr5 | 142780531 | 5'UTR | -0.052 [-0.165 – 0.061] | 0.59 |  |  |  |  |
|  | cg08845721 | chr5 | 142780693 | 5'UTR | 0.042 [-0.094 – 0.177] | 0.76 |  |  |  |  |
|  | cg12969488 | chr5 | 142780984 | 5'UTR | 0.053 [-0.028 – 0.134] | 0.42 |  |  |  |  |
|  | cg07733851 | chr5 | 142781498 | 5'UTR | -0.095 [-0.196 – 0.005] | 0.24 |  |  | 5:142781453-142781534 |  |
|  | cg18998365 | chr5 | 142781532 | 5'UTR | -0.084 [-0.182 – 0.014] | 0.29 |  |  | 5:142781453-142781534 |  |
|  | cg27122725 | chr5 | 142781723 | 5'UTR | -0.021 [-0.106 – 0.063] | 0.79 |  |  | 5:142781700-142781770 |  |
|  | cg06952416 | chr5 | 142781736 | 5'UTR | -0.015 [-0.082 – 0.052] | 0.81 |  |  | 5:142781700-142781770 |  |
|  | cg06521673 | chr5 | 142782072 | 5'UTR | 0.024 [-0.014 – 0.063] | 0.45 |  |  |  |  |
|  | cg17617527 | chr5 | 142782415 | 5'UTR | 0.014 [-0.009 – 0.037] | 0.45 |  |  |  |  |
|  | cg20753294 | chr5 | 142782791 | 5'UTR/1stExon | 0.040 [-0.042 – 0.122] | 0.56 | high-CpG:142762947-142763503 |  |  |  |
|  | cg18146873 | chr5 | 142782827 | 5'UTR/1stExon | 0.029 [-0.014 – 0.073] | 0.41 | high-CpG:142762947-142763503 |  |  |  |
|  | cg00629244 | chr5 | 142783379 | Promoter/5'UTR | 0.012 [-0.010 – 0.033] | 0.53 |  |  | 5:142783021-142785530 |  |
|  | cg11152298 | chr5 | 142783383 | Promoter/5'UTR | -0.022 [-0.056 – 0.012] | 0.43 |  |  | 5:142783021-142785530 |  |
|  | cg18019515 | chr5 | 142783385 | Promoter/5'UTR | 0.025 [-0.019 – 0.070] | 0.50 |  |  | 5:142783021-142785530 |  |
|  | cg17860381 | chr5 | 142783569 | Promoter/5'UTR | 0.054 [0.005 – 0.104] | 0.16 | high-CpG:142763715-142763897 |  | 5:142783021-142785530 |  |
|  | cg04111177 | chr5 | 142783607 | Promoter/5'UTR | -0.002 [-0.042 – 0.038] | 0.96 | high-CpG:142763715-142763897 |  | 5:142783021-142785530 |  |
|  | cg15910486 | chr5 | 142783621 | Promoter/5'UTR | 0.090 [0.039 – 0.141] | 0.034 | high-CpG:142763715-142763897 |  | 5:142783021-142785530 |  |
|  | cg15645634 | chr5 | 142783639 | Promoter/5'UTR | 0.002 [-0.021 – 0.025] | 0.92 | high-CpG:142763715-142763897 |  | 5:142783021-142785530 |  |
|  | cg14939152 | chr5 | 142783831 | Promoter/5'UTR | 0.024 [-0.003 – 0.050] | 0.26 | high-CpG:142763995-142764317 |  | 5:142783021-142785530 |  |
|  | cg18068240 | chr5 | 142783843 | Promoter/5'UTR | 0.011 [-0.014 – 0.036] | 0.63 | high-CpG:142763995-142764317 |  | 5:142783021-142785530 |  |
|  | cg21209684 | chr5 | 142783848 | Promoter/5'UTR | 0.034 [0.010 – 0.058] | 0.070 | high-CpG:142763995-142764317 |  | 5:142783021-142785530 |  |
|  | cg01967637 | chr5 | 142784019 | Promoter/5'UTR/1stExon | 0.100 [0.037 – 0.163] | 0.050 | high-CpG:142763995-142764317 |  | 5:142783021-142785530 |  |
|  | cg22402730 | chr5 | 142784168 | Promoter/5'UTR | -0.023 [-0.052 – 0.006] | 0.33 |  |  | 5:142783021-142785530 |  |
|  | cg19135245 | chr5 | 142784187 | Promoter/5'UTR | 0.021 [-0.015 – 0.056] | 0.50 |  |  | 5:142783021-142785530 |  |
|  | cg26464411 | chr5 | 142784222 | Promoter/5'UTR | 0.046 [-0.021 – 0.113] | 0.41 |  |  | 5:142783021-142785530 |  |
|  | cg07515400 | chr5 | 142784278 | Promoter/5'UTR | 0.003 [-0.045 – 0.051] | 0.94 |  |  | 5:142783021-142785530 |  |
|  | cg06968181 | chr5 | 142784323 | Promoter/5'UTR | 0.006 [-0.035 – 0.047] | 0.86 |  |  | 5:142783021-142785530 |  |
|  | cg18849621 | chr5 | 142784382 | Promoter/5'UTR | -0.023 [-0.067 – 0.021] | 0.54 |  |  | 5:142783021-142785530 |  |
|  | cg16335926 | chr5 | 142784462 | Promoter/5'UTR | 0.004 [-0.049 – 0.057] | 0.93 |  |  | 5:142783021-142785530 |  |
|  | cg10847032 | chr5 | 142784522 | Promoter/5'UTR | -0.025 [-0.059 – 0.009] | 0.36 |  |  | 5:142783021-142785530 |  |
|  | cg21702128 | chr5 | 142784721 | Promoter/5'UTR | 0.021 [-0.011 – 0.053] | 0.43 |  |  | 5:142783021-142785530 |  |
|  | cg14558428 | chr5 | 142784982 | Promoter/5'UTR | 0.019 [-0.005 – 0.044] | 0.33 |  |  | 5:142783021-142785530 |  |
|  | cg24026230 | chr5 | 142785172 | Promoter/5'UTR | 0.036 [0.004 – 0.068] | 0.15 |  |  | 5:142783021-142785530 |  |
|  | cg13648501 | chr5 | 142785258 | Promoter/5'UTR | 0.011 [-0.026 – 0.048] | 0.78 |  |  | 5:142783021-142785530 |  |
|  | cg18718518 | chr5 | 142785455 | Promoter/5'UTR | 0.004 [-0.024 – 0.032] | 0.88 |  | chr5:142785420-142785420 | 5:142783021-142785530 |  |
|  | cg13764763 | chr5 | 142785501 | Promoter/5'UTR | 0.022 [-0.036 – 0.080] | 0.69 |  | chr5:142785420-142785420 | 5:142783021-142785530 |  |
|  | cg27345592 | chr5 | 142786405 | 5'UTR | -0.016 [-0.057 – 0.024] | 0.67 |  |  |  |  |
|  | cg07528216 | chr5 | 142788776 | 5'UTR | -0.008 [-0.038 – 0.023] | 0.79 |  |  |  |  |
|  | cg16224829 | chr5 | 142792698 | 5'UTR | -0.051 [-0.097 – -0.005] | 0.16 |  |  |  |  |
|  | cg01751279 | chr5 | 142793924 | 5'UTR | -0.015 [-0.055 – 0.025] | 0.69 |  |  | 5:142793737-142794054 |  |
|  | cg23430507 | chr5 | 142798375 | 5'UTR | -0.001 [-0.083 – 0.080] | 0.98 |  |  |  |  |
|  | cg17349736 | chr5 | 142802329 | 5'UTR | -0.005 [-0.041 – 0.031] | 0.87 |  |  |  |  |
|  | cg14438279 | chr5 | 142806343 | 5'UTR | -0.034 [-0.071 – 0.003] | 0.25 |  |  |  |  |
|  | cg25579735 | chr5 | 142807171 | 5'UTR | 0.000 [-0.035 – 0.034] | 0.99 |  |  |  |  |
|  | cg08423118 | chr5 | 142808610 | 5'UTR | -0.040 [-0.076 – -0.004] | 0.15 |  |  |  |  |
|  | cg23776787 | chr5 | 142814315 | 5'UTR/1stExon | -0.061 [-0.115 – -0.006] | 0.15 |  |  | 5:142813985-142815209 |  |
|  | cg03906910 | chr5 | 142814388 | 5'UTR/1stExon | -0.142 [-0.246 – -0.038] | 0.078 |  |  | 5:142813985-142815209 |  |
|  | cg08818984 | chr5 | 142814827 | 5'UTR/1stExon | -0.096 [-0.188 – -0.003] | 0.19 |  |  | 5:142813985-142815209 |  |
|  | cg26720913 | chr5 | 142814934 | 5'UTR/1stExon | -0.163 [-0.265 – -0.062] | 0.050 |  |  | 5:142813985-142815209 |  |
|  | cg07589972 | chr5 | 142815463 | Promoter | -0.007 [-0.036 – 0.023] | 0.81 |  |  |  |  |
|  | cg12466613 | chr5 | 142815469 | Promoter | 0.015 [-0.033 – 0.064] | 0.75 |  |  |  |  |
|  | cg21979215 | chr5 | 142815807 | Promoter | -0.066 [-0.173 – 0.041] | 0.46 |  |  |  |  |
|  | cg01294526 | chr5 | 142816417 | Promoter | -0.001 [-0.047 – 0.045] | 0.98 |  |  |  |  |
| HSD11B1 | cg12065670 | chr1 | 209858339 | Promoter | 0.024 [-0.025 – 0.073] | 0.57 |  |  |  |  |
|  | cg04732193 | chr1 | 209858581 | Promoter | -0.012 [-0.046 – 0.023] | 0.72 |  |  |  |  |
|  | cg12761518 | chr1 | 209858806 | Promoter | -0.021 [-0.054 – 0.012] | 0.45 |  |  |  |  |
|  | cg22248789 | chr1 | 209858842 | Promoter | -0.024 [-0.050 – 0.002] | 0.25 |  |  |  |  |
|  | cg03996090 | chr1 | 209859331 | Promoter | -0.091 [-0.156 – -0.026] | 0.070 |  |  |  |  |
|  | cg10921886 | chr1 | 209859350 | Promoter | -0.096 [-0.159 – -0.033] | 0.052 |  |  |  |  |
|  | cg06828613 | chr1 | 209859738 | 5'UTR | -0.031 [-0.075 – 0.013] | 0.40 |  |  |  |  |
|  | cg25471557 | chr1 | 209862480 | 5'UTR | -0.005 [-0.056 – 0.047] | 0.92 |  |  |  |  |
|  | cg17726275 | chr1 | 209865574 | 5'UTR | -0.011 [-0.047 – 0.025] | 0.76 |  |  |  |  |
|  | cg05401402 | chr1 | 209870867 | 5'UTR | -0.006 [-0.046 – 0.033] | 0.86 |  |  |  |  |
|  | cg16897216 | chr1 | 209876837 | Promoter/5'UTR | -0.029 [-0.064 – 0.007] | 0.32 |  |  |  |  |
|  | cg14139038 | chr1 | 209877340 | Promoter/5'UTR | -0.078 [-0.118 – -0.039] | 0.0098 |  |  |  |  |
|  | cg20191437 | chr1 | 209877382 | Promoter/5'UTR | -0.035 [-0.071 – 0.001] | 0.22 |  |  |  |  |
|  | cg00312486 | chr1 | 209877438 | Promoter/5'UTR | -0.027 [-0.065 – 0.010] | 0.38 |  |  |  |  |
|  | cg26880525 | chr1 | 209877941 | Promoter/5'UTR | -0.001 [-0.037 – 0.034] | 0.96 |  |  |  |  |
|  | cg09366519 | chr1 | 209877970 | Promoter/5'UTR | -0.052 [-0.101 – -0.003] | 0.19 |  |  |  |  |
|  | cg05112220 | chr1 | 209878097 | Promoter/5'UTR | -0.115 [-0.200 – -0.030] | 0.078 |  |  |  |  |
|  | cg06571187 | chr1 | 209878123 | Promoter/5'UTR | -0.108 [-0.177 – -0.039] | 0.050 |  |  |  |  |
|  | cg04484523 | chr1 | 209878389 | Body | -0.021 [-0.065 – 0.024] | 0.59 |  |  |  |  |
|  | cg11926430 | chr1 | 209883607 | Body | -0.019 [-0.058 – 0.019] | 0.56 |  |  |  |  |
|  | cg17610535 | chr1 | 209897614 | Body | 0.030 [-0.030 – 0.090] | 0.56 |  |  |  |  |
|  | cg02947427 | chr1 | 209897658 | Body | 0.001 [-0.020 – 0.039] | 0.75 |  |  |  |  |
|  | cg08780327 | chr1 | 209897837 | Body | 0.054 [0.014 – 0.095] | 0.081 |  |  | 1:209897750-209898004 |  |
|  | cg17297831 | chr1 | 209898505 | Body | -0.040 [-0.081 – 0.001] | 0.22 |  |  |  |  |
|  | cg27403618 | chr1 | 209907350 | Body | -0.049 [-0.104 – 0.007] | 0.27 |  |  |  |  |
| HSD11B2 | cg04712664 | chr16 | 67463902 | Promoter | 0.101 [0.025 – 0.176] | 0.082 |  |  |  |  |
|  | cg15690037 | chr16 | 67464093 | Promoter | 0.096 [0.030 – 0.162] | 0.060 |  |  | 16:67463960-67465526 |  |
|  | cg27130954 | chr16 | 67464331 | Promoter | 0.046 [0.004 – 0.088] | 0.16 |  |  | 16:67463960-67465526 |  |
|  | cg08711598 | chr16 | 67464483 | Promoter | 0.065 [0.012 – 0.117] | 0.11 |  |  | 16:67463960-67465526 |  |
|  | cg02955911 | chr16 | 67464503 | Promoter | 0.022 [0.000 – 0.045] | 0.22 | high-CpG:66021987-66022080 |  | 16:67463960-67465526 |  |
|  | cg16545496 | chr16 | 67464522 | Promoter | 0.018 [-0.006 – 0.043] | 0.34 | high-CpG:66021987-66022080 |  | 16:67463960-67465526 |  |
|  | cg02322203 | chr16 | 67464610 | Promoter | 0.028 [-0.002 – 0.058] | 0.24 | high-CpG:66022066-66022469 |  | 16:67463960-67465526 |  |
|  | cg08789908 | chr16 | 67464707 | Promoter | -0.003 [-0.041 – 0.035] | 0.92 | high-CpG:66022066-66022469 |  | 16:67463960-67465526 |  |
|  | cg07545640 | chr16 | 67464829 | Promoter | 0.011 [-0.01 – 0.031] | 0.54 | high-CpG:66022066-66022469 |  | 16:67463960-67465526 |  |
|  | cg12790440 | chr16 | 67464868 | Promoter | -0.023 [-0.082 – 0.036] | 0.68 | high-CpG:66022066-66022469 |  | 16:67463960-67465526 |  |
|  | cg10686375 | chr16 | 67464878 | Promoter | 0.006 [-0.028 – 0.041] | 0.85 | high-CpG:66022066-66022469 |  | 16:67463960-67465526 |  |
|  | cg13753571 | chr16 | 67464886 | Promoter | 0.067 [0.003 – 0.131] | 0.19 | high-CpG:66022066-66022469 |  | 16:67463960-67465526 |  |
|  | cg20556751 | chr16 | 67464891 | Promoter | 0.036 [-0.005 – 0.078] | 0.28 | high-CpG:66022066-66022469 |  | 16:67463960-67465526 |  |
|  | cg24270678 | chr16 | 67465029 | Promoter | 0.024 [-0.007 – 0.055] | 0.34 | high-CpG:66022520-66022749 |  | 16:67463960-67465526 |  |
|  | cg12670061 | chr16 | 67465042 | 5'UTR/1stExon | 0.016 [-0.004 – 0.037] | 0.33 | high-CpG:66022520-66022749 |  | 16:67463960-67465526 |  |
|  | cg07724674 | chr16 | 67465455 | Body | 0.007 [-0.031 – 0.045] | 0.84 |  |  | 16:67463960-67465526 |  |
|  | cg20981893 | chr16 | 67465461 | Body | 0.044 [0.002 – 0.087] | 0.19 |  |  | 16:67463960-67465526 |  |
|  | cg12081455 | chr16 | 67465561 | Body | 0.090 [0.021 – 0.159] | 0.084 |  |  |  |  |
|  | cg01443318 | chr16 | 67466260 | Body | -0.014 [-0.055 – 0.027] | 0.72 |  |  | 16:67465852-67466435 |  |
|  | cg13678281 | chr16 | 67466470 | Body | -0.016 [-0.048 – 0.015] | 0.55 |  |  |  |  |
|  | cg02734600 | chr16 | 67467751 | Body | -0.010 [-0.039 – 0.018] | 0.70 |  |  |  |  |
|  | cg09807841 | chr16 | 67469937 | Body | -0.004 [-0.053 – 0.045] | 0.92 |  |  |  |  |
|  | cg04750517 | chr16 | 67471172 | 3'UTR | -0.008 [-0.036 – 0.021] | 0.79 |  |  |  |  |
| SRD5A1 | cg26420013 | chr5 | 6632020 | Promoter | -0.077 [-0.128 – -0.025] | 0.052 |  |  |  |  |
|  | cg22911074 | chr5 | 6632973 | Promoter | 0.081 [0.031 – 0.130] | 0.049 |  |  | 5:6632952-6633655 |  |
|  | cg01541629 | chr5 | 6633111 | Promoter | -0.057 [-0.139 – 0.026] | 0.41 | high-CpG:6686105-6686448 |  | 5:6632952-6633655 |  |
|  | cg17641279 | chr5 | 6633215 | Promoter | 0.021 [-0.018 – 0.060] | 0.54 | high-CpG:6686105-6686448 |  | 5:6632952-6633655 |  |
|  | cg01395659 | chr5 | 6633232 | Promoter | 0.012 [-0.015 – 0.039] | 0.61 | high-CpG:6686105-6686448 |  | 5:6632952-6633655 |  |
|  | cg07758902 | chr5 | 6633253 | Promoter | -0.009 [-0.048 – 0.031] | 0.81 | high-CpG:6686105-6686448 |  | 5:6632952-6633655 |  |
|  | cg15879084 | chr5 | 6633297 | Promoter | 0.017 [-0.013 – 0.048] | 0.50 | high-CpG:6686105-6686448 |  | 5:6632952-6633655 |  |
|  | cg16857840 | chr5 | 6633481 | Promoter | 0.023 [-0.007 – 0.052] | 0.34 |  |  | 5:6632952-6633655 |  |
|  | cg25394652 | chr5 | 6633491 | Promoter | 0.039 [-0.01 – 0.088] | 0.33 |  |  | 5:6632952-6633655 |  |
|  | cg07094875 | chr5 | 6633508 | 5'UTR/1stExon | 0.026 [-0.013 – 0.066] | 0.42 | high-CpG:6686502-6686683 |  | 5:6632952-6633655 |  |
|  | cg23242142 | chr5 | 6633531 | 5'UTR/1stExon | 0.015 [-0.048 – 0.079] | 0.79 | high-CpG:6686502-6686683 |  | 5:6632952-6633655 |  |
|  | cg10459717 | chr5 | 6633577 | 5'UTR/1stExon | -0.013 [-0.046 – 0.021] | 0.69 | high-CpG:6686502-6686683 |  | 5:6632952-6633655 |  |
|  | cg16431963 | chr5 | 6634117 | Body | 0.011 [-0.029 – 0.051] | 0.79 |  |  |  |  |
|  | cg16742195 | chr5 | 6634300 | Body | -0.001 [-0.038 – 0.037] | 0.98 |  |  |  |  |
|  | cg10408813 | chr5 | 6634308 | Body | -0.008 [-0.044 – 0.027] | 0.79 |  |  |  |  |
|  | cg19229903 | chr5 | 6634932 | Body | 0.150 [0.043 – 0.257] | 0.070 |  |  |  |  |
|  | cg08700190 | chr5 | 6636046 | Body | 0.201 [0.054 – 0.348] | 0.078 |  |  | 5:6635992-6636200 |  |
|  | cg23974739 | chr5 | 6637525 | Body | -0.086 [-0.151 – -0.021] | 0.082 |  |  |  |  |
|  | cg17774776 | chr5 | 6638536 | Body | 0.155 [0.023 – 0.287] | 0.13 |  |  |  |  |
|  | cg14328737 | chr5 | 6645364 | Body | -0.076 [-0.135 – -0.018] | 0.084 |  |  |  |  |
|  | cg21001997 | chr5 | 6652146 | Body | -0.011 [-0.052 – 0.031] | 0.79 |  |  |  |  |
|  | cg10549357 | chr5 | 6653742 | Body | -0.011 [-0.047 – 0.025] | 0.76 |  |  |  |  |
|  | cg17772763 | chr5 | 6659801 | Body | -0.044 [-0.102 – 0.013] | 0.34 |  |  |  |  |
|  | cg16832396 | chr5 | 6660096 | Body | -0.017 [-0.049 – 0.016] | 0.55 |  |  |  |  |
|  | cg08881505 | chr5 | 6660406 | Body | -0.009 [-0.069 – 0.051] | 0.87 |  |  | 5:6660332-6660766 |  |
|  | cg06879410 | chr5 | 6664808 | Body | -0.027 [-0.067 – 0.014] | 0.43 |  |  |  |  |
|  | cg18755984 | chr5 | 6667258 | Body | -0.007 [-0.060 – 0.045] | 0.88 |  |  |  |  |
|  | cg08258978 | chr5 | 6667276 | Body | -0.016 [-0.058 – 0.026] | 0.70 |  |  |  |  |
|  | cg11469319 | chr5 | 6667352 | Body | 0.005 [-0.031 – 0.041] | 0.88 |  |  |  |  |
|  | cg05879499 | chr5 | 6668385 | 3'UTR | -0.026 [-0.073 – 0.021] | 0.51 |  |  |  |  |
|  | cg08933496 | chr5 | 6669208 | 3'UTR | 0.014 [-0.030 – 0.058] | 0.75 |  |  |  |  |
| SRD5A2 | cg13181695 | chr2 | 31749899 | 3'UTR | 0.033 [-0.016 – 0.082] | 0.42 |  |  |  |  |
|  | cg09728179 | chr2 | 31749988 | 3'UTR | -0.015 [-0.048 – 0.018] | 0.61 |  |  |  |  |
|  | cg25062017 | chr2 | 31750589 | 3'UTR | -0.034 [-0.064 – -0.004] | 0.14 |  |  |  |  |
|  | cg20923628 | chr2 | 31750968 | 3'UTR | -0.015 [-0.054 – 0.024] | 0.69 |  |  |  |  |
|  | cg06893549 | chr2 | 31751294 | Body | -0.020 [-0.050 – 0.011] | 0.44 |  |  |  |  |
|  | cg19437905 | chr2 | 31751826 | Body | 0.005 [-0.025 – 0.035] | 0.86 |  |  |  |  |
|  | cg27329573 | chr2 | 31757394 | Body | 0.052 [-0.011 – 0.115] | 0.31 |  |  |  |  |
|  | cg06914213 | chr2 | 31784009 | Body | -0.071 [-0.142 – -0.001] | 0.20 |  |  |  |  |
|  | cg02716041 | chr2 | 31788243 | Body | 0.006 [-0.059 – 0.071] | 0.92 |  |  |  |  |
|  | cg06036196 | chr2 | 31802462 | Body | -0.024 [-0.060 – 0.012] | 0.42 |  |  |  |  |
|  | cg04900872 | chr2 | 31803536 | Body | -0.017 [-0.083 – 0.050] | 0.79 |  |  |  |  |
|  | cg14174946 | chr2 | 31805218 | Body | 0.041 [-0.003 – 0.085] | 0.24 |  |  |  |  |
|  | cg19502744 | chr2 | 31805915 | 1stExon | 0.031 [-0.023 – 0.085] | 0.50 |  |  |  |  |
|  | cg18948125 | chr2 | 31805970 | 5'UTR/1stExon | -0.020 [-0.055 – 0.015] | 0.51 |  |  |  |  |
|  | cg18529845 | chr2 | 31806042 | Promoter | 0.008 [-0.023 – 0.040] | 0.79 |  |  |  |  |
|  | cg26261627 | chr2 | 31806173 | Promoter | -0.015 [-0.127 – 0.098] | 0.89 |  |  |  |  |
|  | cg26638505 | chr2 | 31806183 | Promoter | -0.004 [-0.094 – 0.087] | 0.96 |  |  |  |  |
|  | cg08894761 | chr2 | 31806234 | Promoter | -0.040 [-0.132 – 0.052] | 0.62 |  |  |  |  |
|  | cg15403517 | chr2 | 31806275 | Promoter | -0.041 [-0.143 – 0.060] | 0.66 |  |  |  |  |
|  | cg02049734 | chr2 | 31806284 | Promoter | -0.004 [-0.046 – 0.038] | 0.91 |  |  |  |  |
|  | cg06492144 | chr2 | 31806352 | Promoter | -0.028 [-0.084 – 0.029] | 0.56 |  |  |  |  |
|  | cg11765362 | chr2 | 31806551 | Promoter | -0.086 [-0.196 – 0.024] | 0.33 |  |  |  |  |
|  | cg06149357 | chr2 | 31806755 | Promoter | -0.029 [-0.087 – 0.029] | 0.56 |  |  |  |  |
|  | cg13527387 | chr2 | 31806767 | Promoter | -0.012 [-0.074 – 0.050] | 0.84 |  |  |  |  |
|  | cg00712943 | chr2 | 31806781 | Promoter | -0.019 [-0.091 – 0.054] | 0.79 |  |  |  |  |
|  | cg22549881 | chr2 | 31806898 | Promoter | -0.020 [-0.121 – 0.082] | 0.84 |  |  |  |  |
|  | cg17267976 | chr2 | 31807474 | Promoter | -0.016 [-0.058 – 0.026] | 0.69 |  |  |  |  |
| AKR1D1 | cg06696666 | chr7 | 137759961 | Promoter | -0.096 [-0.200 – 0.008] | 0.25 |  |  |  |  |
|  | cg25271841 | chr7 | 137760128 | Promoter | -0.038 [-0.067 – -0.009] | 0.082 |  |  |  |  |
|  | cg09277575 | chr7 | 137760222 | Promoter | -0.028 [-0.074 – 0.018] | 0.46 |  |  |  |  |
|  | cg20518096 | chr7 | 137760331 | Promoter | -0.007 [-0.055 – 0.040] | 0.86 |  |  |  |  |
|  | cg15002691 | chr7 | 137761047 | Promoter | 0.004 [-0.050 – 0.058] | 0.92 |  |  |  |  |
|  | cg20647137 | chr7 | 137761289 | 1stExon | 0.005 [-0.024 – 0.035] | 0.85 |  |  |  |  |
|  | cg09373725 | chr7 | 137774429 | Body | -0.098 [-0.159 – -0.038] | 0.049 |  |  |  |  |
|  | cg17257469 | chr7 | 137775104 | Body | 0.071 [0.004 – 0.138] | 0.18 |  |  |  |  |
|  | cg16333323 | chr7 | 137775592 | Body | -0.026 [-0.076 – 0.024] | 0.55 |  |  |  |  |
|  | cg27250318 | chr7 | 137775797 | Body | -0.181 [-0.290 – -0.071] | 0.049 |  |  |  |  |
|  | cg05082563 | chr7 | 137775882 | Body | -0.127 [-0.208 – -0.046] | 0.050 |  |  |  |  |
|  | cg11110544 | chr7 | 137776547 | Body | -0.020 [-0.063 – 0.023] | 0.59 |  |  |  |  |
|  | cg02118020 | chr7 | 137782462 | Body | 0.188 [0.067 – 0.310] | 0.050 |  |  |  |  |
|  | cg26317963 | chr7 | 137801370 | Body | 0.003 [-0.025 – 0.031] | 0.91 |  |  |  |  |
|  | cg24212267 | chr7 | 137801780 | 3'UTR | 0.034 [-0.037 – 0.106] | 0.57 |  |  |  |  |
| DUSP1 | cg02029908 | chr5 | 172195602 | 3'UTR | 0.262 [0.068 – 0.456] | 0.079 |  |  |  |  |
|  | cg07018389 | chr5 | 172195678 | 3'UTR | 0.113 [0.012 – 0.214] | 0.15 |  |  |  |  |
|  | cg00593243 | chr5 | 172196153 | Body | 0.184 [0.071 – 0.297] | 0.049 |  |  | 5:172196108-172196195 |  |
|  | cg17378966 | chr5 | 172196746 | Body | 0.204 [0.043 – 0.366] | 0.095 |  |  |  |  |
|  | cg15890707 | chr5 | 172197278 | Body | 0.043 [-0.011 – 0.098] | 0.33 |  |  |  |  |
|  | cg08293091 | chr5 | 172197350 | Body | -0.001 [-0.040 – 0.038] | 0.98 |  |  |  |  |
|  | cg26095194 | chr5 | 172197489 | Body | 0.022 [-0.015 – 0.059] | 0.48 |  |  |  |  |
|  | cg17429424 | chr5 | 172197911 | 1stExon | -0.028 [-0.069 – 0.013] | 0.42 |  |  | 5:172197527-172198566 |  |
|  | cg22229034 | chr5 | 172198031 | 5'UTR/1stExon | 0.029 [0.001 – 0.057] | 0.20 |  |  | 5:172197527-172198566 |  |
|  | cg17946497 | chr5 | 172198141 | 5'UTR/1stExon | 0.050 [0.001 – 0.099] | 0.20 |  |  | 5:172197527-172198566 |  |
|  | cg19801141 | chr5 | 172198162 | 5'UTR/1stExon | 0.017 [-0.009 – 0.043] | 0.43 | high-CpG:172130766-172131260 |  | 5:172197527-172198566 |  |
|  | cg27222301 | chr5 | 172198261 | Promoter | 0.024 [-0.002 – 0.051] | 0.25 | high-CpG:172130766-172131260 |  | 5:172197527-172198566 |  |
|  | cg07013334 | chr5 | 172198263 | Promoter | 0.019 [-0.013 – 0.051] | 0.49 | high-CpG:172130766-172131260 |  | 5:172197527-172198566 |  |
|  | cg11757894 | chr5 | 172198326 | Promoter | 0.025 [-0.014 – 0.063] | 0.43 | high-CpG:172130766-172131260 |  | 5:172197527-172198566 |  |
|  | cg04525476 | chr5 | 172198714 | Promoter | 0.020 [-0.016 – 0.056] | 0.53 |  |  | 5:172198628-172199895 |  |
|  | cg12884779 | chr5 | 172198737 | Promoter | 0.073 [-0.009 – 0.155] | 0.27 |  |  | 5:172198628-172199895 |  |
|  | cg04060275 | chr5 | 172198791 | Promoter | 0.019 [-0.009 – 0.048] | 0.42 |  | chr5:172198749-172198749 | 5:172198628-172199895 |  |
|  | cg05612977 | chr5 | 172198876 | Promoter | 0.032 [0.006 – 0.057] | 0.099 |  | chr5:172198749-172198749 | 5:172198628-172199895 |  |
|  | cg15414042 | chr5 | 172198979 | Promoter | 0.046 [0.000 – 0.092] | 0.21 |  | chr5:172198749-172198749 | 5:172198628-172199895 |  |
|  | cg18121420 | chr5 | 172199013 | Promoter | 0.019 [-0.018 – 0.056] | 0.55 |  | chr5:172198749-172198749 | 5:172198628-172199895 |  |
|  | cg02352687 | chr5 | 172199101 | Promoter | 0.041 [-0.029 – 0.112] | 0.49 |  | chr5:172198749-172198749 | 5:172198628-172199895 |  |
|  | cg10672508 | chr5 | 172199157 | Promoter | 0.022 [-0.003 – 0.046] | 0.27 |  | chr5:172198749-172198749 | 5:172198628-172199895 |  |
|  | cg10837843 | chr5 | 172199244 | Promoter | 0.001 [-0.036 – 0.038] | 0.97 |  | chr5:172198749-172198749 | 5:172198628-172199895 |  |
|  | cg23162310 | chr5 | 172199255 | Promoter | 0.001 [-0.034 – 0.036] | 0.96 |  | chr5:172198749-172198749 | 5:172198628-172199895 |  |
|  | cg06819445 | chr5 | 172199278 | Promoter | 0.011 [-0.031 – 0.053] | 0.79 |  | chr5:172198749-172198749 | 5:172198628-172199895 |  |
|  | cg12333707 | chr5 | 172199303 | Promoter | -0.009 [-0.047 – 0.029] | 0.79 |  | chr5:172198749-172198749 | 5:172198628-172199895 |  |
|  | cg25108022 | chr5 | 172199313 | Promoter | 0.074 [0.016 – 0.132] | 0.091 |  | chr5:172198749-172198749 | 5:172198628-172199895 |  |
|  | cg23002268 | chr5 | 172199318 | Promoter | 0.018 [-0.002 – 0.039] | 0.27 |  | chr5:172198749-172198749 | 5:172198628-172199895 |  |
|  | cg19537645 | chr5 | 172199418 | Promoter | 0.012 [-0.011 – 0.034] | 0.55 |  | chr5:172198749-172198749 | 5:172198628-172199895 |  |
|  | cg09799633 | chr5 | 172199460 | Promoter | 0.012 [-0.010 – 0.034] | 0.51 |  | chr5:172198749-172198749 | 5:172198628-172199895 |  |
|  | cg09493150 | chr5 | 172199605 | Promoter | -0.013 [-0.056 – 0.030] | 0.76 |  | chr5:172198749-172198749 | 5:172198628-172199895 |  |
|  | cg16957313 | chr5 | 172199661 | Promoter | -0.105 [-0.192 – -0.019] | 0.11 |  | chr5:172198749-172198749 | 5:172198628-172199895 |  |
|  | cg22473727 | chr5 | 172199667 | Promoter | -0.121 [-0.202 – -0.040] | 0.052 |  | chr5:172198749-172198749 | 5:172198628-172199895 |  |
| ANXA1 | cg10822986 | chr9 | 75765398 | Promoter | -0.020 [-0.073 – 0.033] | 0.69 |  |  |  |  |
|  | cg01894895 | chr9 | 75766294 | Promoter | 0.012 [-0.038 – 0.061] | 0.80 |  |  |  |  |
|  | cg13591783 | chr9 | 75768868 | 5'UTR | 0.011 [-0.031 – 0.053] | 0.79 |  |  |  |  |
|  | cg21222681 | chr9 | 75773640 | Body | 0.009 [-0.045 – 0.063] | 0.86 |  |  |  |  |
|  | cg10438252 | chr9 | 75774298 | Body | 0.064 [-0.027 – 0.155] | 0.40 |  |  |  |  |
|  | cg06231148 | chr9 | 75782700 | Body | -0.054 [-0.111 – 0.004] | 0.24 |  |  |  |  |
| PCSK1 | cg20604493 | chr5 | 95738023 | Body | -0.009 [-0.043 – 0.025] | 0.79 |  |  |  |  |
|  | cg21582513 | chr5 | 95748708 | Body | 0.085 [-0.002 – 0.172] | 0.22 |  |  |  |  |
|  | cg08153016 | chr5 | 95748963 | Body | 0.098 [-0.018 – 0.213] | 0.30 |  |  |  |  |
|  | cg04477939 | chr5 | 95748998 | Body | 0.194 [0.034 – 0.355] | 0.11 |  |  |  |  |
|  | cg20435994 | chr5 | 95750057 | Body | -0.020 [-0.046 – 0.006] | 0.33 |  |  |  |  |
|  | cg22824890 | chr5 | 95763787 | Body | -0.052 [-0.107 – 0.002] | 0.23 |  |  |  |  |
|  | cg19084281 | chr5 | 95765746 | Body | 0.019 [-0.060 – 0.099] | 0.79 |  |  |  |  |
|  | cg10607455 | chr5 | 95767550 | Body | -0.056 [-0.134 – 0.023] | 0.39 |  |  |  |  |
|  | cg21878859 | chr5 | 95767724 | Body | 0.028 [-0.027 – 0.084] | 0.55 |  |  |  |  |
|  | cg10988336 | chr5 | 95767768 | Body | 0.030 [-0.020 – 0.080] | 0.46 |  |  |  |  |
|  | cg05844847 | chr5 | 95767842 | 5'UTR/1stExon/Body | 0.010 [-0.043 – 0.063] | 0.84 |  |  |  |  |
|  | cg13912090 | chr5 | 95767863 | Body | -0.036 [-0.090 – 0.018] | 0.42 |  |  |  |  |
|  | cg22873959 | chr5 | 95767928 | Promoter/Body | -0.028 [-0.065 – 0.010] | 0.36 |  |  |  |  |
|  | cg16949465 | chr5 | 95767946 | Promoter/Body | -0.005 [-0.042 – 0.033] | 0.89 |  |  |  |  |
|  | cg03763570 | chr5 | 95768016 | Promoter/Body | 0.041 [0.005 – 0.078] | 0.15 |  |  |  |  |
|  | cg14339134 | chr5 | 95768053 | Promoter/Body | 0.016 [-0.035 – 0.068] | 0.75 |  |  |  |  |
|  | cg19456953 | chr5 | 95768418 | Body | 0.015 [-0.046 – 0.076] | 0.79 |  |  |  |  |
|  | cg09786257 | chr5 | 95768695 | 1stExon | -0.014 [-0.044 – 0.015] | 0.57 |  |  |  |  |
|  | cg00637687 | chr5 | 95768915 | 5'UTR/1stExon | 0.000 [-0.045 – 0.046] | 0.99 |  |  |  |  |
|  | cg00959249 | chr5 | 95768997 | Promoter | -0.003 [-0.087 – 0.082] | 0.97 |  |  |  |  |
|  | cg18840956 | chr5 | 95769005 | Promoter | 0.007 [-0.023 – 0.038] | 0.80 |  |  |  |  |
|  | cg23187653 | chr5 | 95769008 | Promoter | -0.014 [-0.082 – 0.054] | 0.83 |  |  |  |  |
|  | cg07145843 | chr5 | 95769025 | Promoter | 0.006 [-0.070 – 0.081] | 0.93 |  |  |  |  |
|  | cg17803175 | chr5 | 95769071 | Promoter | 0.004 [-0.034 – 0.042] | 0.91 |  |  |  |  |
|  | cg02115960 | chr5 | 95769079 | Promoter | -0.022 [-0.060 – 0.015] | 0.48 |  |  |  |  |
|  | cg02015462 | chr5 | 95769172 | Promoter | -0.012 [-0.079 – 0.054] | 0.85 |  |  |  |  |
|  | cg26904823 | chr5 | 95769305 | Promoter | -0.054 [-0.120 – 0.012] | 0.32 |  |  |  |  |
|  | cg01129674 | chr5 | 95769602 | Promoter | -0.010 [-0.052 – 0.031] | 0.79 |  |  |  |  |
|  | cg11348994 | chr5 | 95769993 | Promoter | 0.006 [-0.032 – 0.044] | 0.86 |  |  |  |  |
|  | cg16230720 | chr5 | 95770044 | Promoter | -0.021 [-0.055 – 0.014] | 0.47 |  |  |  |  |
|  | cg05981500 | chr5 | 95770238 | Promoter | 0.009 [-0.030 – 0.048] | 0.81 |  |  |  |  |
| TSC22D3 | cg11837390 | chrX | 106957165 | 3'UTR | -0.003 [-0.054 – 0.049] | 0.95 |  |  | X:106957063-106958012 |  |
|  | cg08117103 | chrX | 106957583 | 3'UTR | -0.017 [-0.072 – 0.037] | 0.75 |  |  | X:106957063-106958012 |  |
|  | cg03915527 | chrX | 106957793 | Body | 0.113 [0.010 – 0.216] | 0.16 |  |  | X:106957063-106958012 |  |
|  | cg21380860 | chrX | 106958499 | Body | 0.059 [-0.026 – 0.145] | 0.41 |  |  | X:106958456-106959107 |  |
|  | cg06774124 | chrX | 106959517 | 5'UTR/Body | 0.024 [-0.037 – 0.085] | 0.69 |  |  | X:106959379-106962296 |  |
|  | cg23660678 | chrX | 106959609 | 5'UTR/1stExon/Body | 0.105 [0.025 – 0.185] | 0.083 |  |  | X:106959379-106962296 | DMR_2 |
|  | cg24318558 | chrX | 106959829 | Promoter/Body | 0.077 [0.009 – 0.145] | 0.15 |  |  | X:106959379-106962296 | DMR_2 |
|  | cg11907074 | chrX | 106959895 | Promoter/Body | 0.114 [0.052 – 0.176] | 0.020 |  |  | X:106959379-106962296 | DMR_2 |
|  | cg19854896 | chrX | 106959898 | Promoter/Body | 0.102 [0.035 – 0.169] | 0.051 |  |  | X:106959379-106962296 | DMR_2 |
|  | cg27311392 | chrX | 106959913 | Promoter/Body | 0.043 [-0.012 – 0.099] | 0.34 |  |  | X:106959379-106962296 | DMR_2 |
|  | cg01802138 | chrX | 106959936 | Promoter/1stExon/Body | 0.069 [0.002 – 0.136] | 0.19 |  |  | X:106959379-106962296 | DMR_2 |
|  | cg07464524 | chrX | 106960206 | Promoter/5'UTR/1stExon/Body | 0.067 [-0.027 – 0.161] | 0.39 | low-CpG:106846838-106846951 |  | X:106959379-106962296 | DMR_2 |
|  | cg11550332 | chrX | 106960273 | Promoter/5'UTR/1stExon/Body | 0.071 [0.001 – 0.140] | 0.20 | low-CpG:106846838-106846951 |  | X:106959379-106962296 | DMR_2 |
|  | cg20085561 | chrX | 106960348 | Promoter/Body | 0.043 [-0.016 – 0.102] | 0.37 |  |  | X:106959379-106962296 |  |
|  | cg05866836 | chrX | 106960378 | Promoter/Body | 0.046 [-0.005 – 0.097] | 0.26 |  |  | X:106959379-106962296 |  |
|  | cg01828434 | chrX | 106960491 | Promoter/Body | 0.082 [0.024 – 0.141] | 0.069 |  |  | X:106959379-106962296 |  |
|  | cg02991082 | chrX | 106961541 | Promoter/Body | -0.006 [-0.054 – 0.042] | 0.89 |  |  | X:106959379-106962296 |  |
|  | cg16953966 | chrX | 106962264 | Body | -0.019 [-0.059 – 0.021] | 0.59 |  |  | X:106959379-106962296 |  |
|  | cg06140906 | chrX | 106964304 | Body | 0.030 [-0.044 – 0.104] | 0.66 |  |  |  |  |
|  | cg06326957 | chrX | 106965628 | Body | -0.007 [-0.041 – 0.028] | 0.84 |  |  |  |  |
|  | cg01030166 | chrX | 106966641 | Body | -0.044 [-0.093 – 0.006] | 0.27 |  |  |  |  |
|  | cg20242796 | chrX | 106970315 | Body | 0.153 [0.012 – 0.295] | 0.16 |  |  |  |  |
|  | cg08118324 | chrX | 106973315 | Body | 0.002 [-0.049 – 0.054] | 0.96 |  |  |  |  |
|  | cg24787262 | chrX | 106974931 | Body | -0.042 [-0.096 – 0.012] | 0.34 |  |  |  |  |
|  | cg26954928 | chrX | 106978259 | Body | -0.099 [-0.161 – -0.037] | 0.050 |  |  |  |  |
|  | cg16615461 | chrX | 106979782 | Body | -0.076 [-0.138 – -0.014] | 0.11 |  |  |  |  |
|  | cg08182225 | chrX | 106983073 | Body | -0.036 [-0.082 – 0.009] | 0.33 |  |  |  |  |
|  | cg06587931 | chrX | 106984142 | Body | -0.035 [-0.080 – 0.011] | 0.34 |  | chrX:106984124-106984124 |  |  |
|  | cg16109419 | chrX | 106984875 | Body | 0.015 [-0.049 – 0.078] | 0.81 |  |  | X:106984768-106985865 |  |
|  | cg09147539 | chrX | 106990678 | Body | 0.056 [-0.015 – 0.127] | 0.33 |  |  |  |  |
|  | cg18396637 | chrX | 107016227 | Body | -0.024 [-0.089 – 0.042] | 0.70 |  |  |  |  |
|  | cg26550835 | chrX | 107017362 | Body | 0.089 [0.007 – 0.171] | 0.17 |  |  |  |  |
|  | cg10982861 | chrX | 107018095 | Body | 0.125 [-0.004 – 0.254] | 0.22 |  |  |  |  |
|  | cg00404599 | chrX | 107018431 | 1stExon | 0.017 [-0.054 – 0.089] | 0.79 |  |  | X:107018416-107018608 |  |
|  | cg07174097 | chrX | 107018607 | 1stExon | 0.052 [-0.012 – 0.116] | 0.32 |  |  | X:107018416-107018608 |  |
|  | cg22823009 | chrX | 107019005 | 5'UTR/1stExon | 0.068 [-0.012 – 0.147] | 0.29 | high-CpG:106905637-106905668 |  | X:107018859-107021211 |  |
|  | cg07251282 | chrX | 107019147 | Promoter | 0.033 [-0.033 – 0.100] | 0.56 |  |  | X:107018859-107021211 |  |
|  | cg21902627 | chrX | 107019166 | Promoter | 0.052 [-0.012 – 0.115] | 0.32 |  |  | X:107018859-107021211 |  |
|  | cg04491396 | chrX | 107019182 | Promoter | 0.081 [0.015 – 0.148] | 0.11 |  |  | X:107018859-107021211 |  |
|  | cg07473550 | chrX | 107019191 | Promoter | 0.055 [-0.014 – 0.124] | 0.33 |  |  | X:107018859-107021211 |  |
|  | cg09724624 | chrX | 107019195 | Promoter | 0.061 [-0.008 – 0.130] | 0.27 |  |  | X:107018859-107021211 |  |
|  | cg03422380 | chrX | 107019244 | Promoter | 0.057 [-0.005 – 0.120] | 0.25 |  |  | X:107018859-107021211 |  |
|  | cg18677034 | chrX | 107019262 | Promoter | 0.085 [0.009 – 0.160] | 0.15 |  |  | X:107018859-107021211 |  |
|  | cg23264750 | chrX | 107019333 | Promoter | 0.096 [0.025 – 0.167] | 0.078 |  |  | X:107018859-107021211 |  |
|  | cg04027004 | chrX | 107019426 | Promoter | 0.107 [0.035 – 0.179] | 0.052 |  |  | X:107018859-107021211 |  |
|  | cg24142775 | chrX | 107019430 | Promoter | 0.115 [0.034 – 0.197] | 0.069 |  |  | X:107018859-107021211 |  |
|  | cg21456313 | chrX | 107019610 | Promoter | 0.018 [-0.045 – 0.081] | 0.78 |  |  | X:107018859-107021211 |  |
|  | cg07115324 | chrX | 107020327 | Promoter | 0.006 [-0.055 – 0.066] | 0.91 |  |  | X:107018859-107021211 |  |
|  | cg20727493 | chrX | 107020439 | Promoter | -0.021 [-0.075 – 0.034] | 0.69 |  |  | X:107018859-107021211 |  |
|  | cg17652025 | chrX | 107020486 | Promoter | -0.046 [-0.106 – 0.014] | 0.34 |  |  | X:107018859-107021211 |  |
| TNF | cg14910524 | chr6 | 31541948 | Promoter | -0.020 [-0.102 – 0.062] | 0.79 |  |  |  |  |
|  | cg08639424 | chr6 | 31542556 | Promoter | 0.007 [-0.037 – 0.050] | 0.86 |  |  |  |  |
|  | cg19978379 | chr6 | 31542671 | Promoter | -0.024 [-0.065 – 0.017] | 0.49 |  |  |  |  |
|  | cg24452282 | chr6 | 31542740 | Promoter | 0.010 [-0.035 – 0.055] | 0.81 |  |  |  |  |
|  | cg11484872 | chr6 | 31543169 | Promoter | -0.014 [-0.059 – 0.031] | 0.76 |  |  | 6:31543103-31543774 |  |
|  | cg21370522 | chr6 | 31543219 | Promoter | -0.074 [-0.148 – 0.001] | 0.21 |  |  | 6:31543103-31543774 | DMR_1 |
|  | cg19648923 | chr6 | 31543266 | Promoter | -0.013 [-0.085 – 0.059] | 0.85 |  |  | 6:31543103-31543774 | DMR_1 |
|  | cg01569083 | chr6 | 31543289 | Promoter | 0.029 [-0.015 – 0.073] | 0.42 |  |  | 6:31543103-31543774 | DMR_1 |
|  | cg03037030 | chr6 | 31543300 | Promoter | 0.042 [-0.004 – 0.087] | 0.25 |  |  | 6:31543103-31543774 | DMR_1 |
|  | cg12681001 | chr6 | 31543540 | 1stExon | -0.054 [-0.107 – -0.001] | 0.20 |  |  | 6:31543103-31543774 | DMR_1 |
|  | cg21222743 | chr6 | 31543545 | 1stExon | -0.037 [-0.093 – 0.020] | 0.43 |  |  | 6:31543103-31543774 | DMR_1 |
|  | cg10717214 | chr6 | 31543557 | 1stExon | -0.018 [-0.064 – 0.029] | 0.69 |  |  | 6:31543103-31543774 | DMR_1 |
|  | cg04425624 | chr6 | 31543565 | 1stExon | -0.077 [-0.128 – -0.027] | 0.051 |  |  | 6:31543103-31543774 | DMR_1 |
|  | cg21467614 | chr6 | 31543638 | 1stExon | -0.057 [-0.095 – -0.019] | 0.052 |  |  | 6:31543103-31543774 | DMR_1 |
|  | cg08553327 | chr6 | 31543647 | 1stExon | -0.062 [-0.099 – -0.025] | 0.049 |  |  | 6:31543103-31543774 | DMR_1 |
|  | cg26729380 | chr6 | 31543655 | 1stExon | -0.052 [-0.085 – -0.019] | 0.050 |  |  | 6:31543103-31543774 | DMR_1 |
|  | cg10650821 | chr6 | 31543686 | 1stExon | -0.059 [-0.090 – -0.029] | 0.012 |  |  | 6:31543103-31543774 | DMR_1 |
|  | cg17741993 | chr6 | 31544694 | Body | 0.021 [-0.066 – 0.108] | 0.79 |  |  | 6:31544432-31544981 |  |
|  | cg01360627 | chr6 | 31544931 | Body | -0.061 [-0.100 – -0.021] | 0.051 |  |  | 6:31544432-31544981 |  |
|  | cg23384708 | chr6 | 31544934 | Body | -0.054 [-0.101 – -0.007] | 0.14 |  |  | 6:31544432-31544981 |  |
|  | cg20477259 | chr6 | 31544960 | Body | -0.022 [-0.067 – 0.022] | 0.56 |  |  | 6:31544432-31544981 |  |
|  | cg15989608 | chr6 | 31545321 | 3'UTR | 0.035 [-0.009 – 0.078] | 0.33 |  |  |  |  |
|  | cg26736341 | chr6 | 31545342 | 3'UTR | 0.032 [-0.002 – 0.066] | 0.24 |  |  |  |  |
|  | cg04472685 | chr6 | 31545473 | 3'UTR | 0.012 [-0.025 – 0.049] | 0.73 |  |  |  |  |
|  | cg19124225 | chr6 | 31545836 | 3'UTR | -0.031 [-0.066 – 0.004] | 0.27 |  |  |  |  |
|  | cg02137984 | chr6 | 31545898 | 3'UTR | -0.016 [-0.047 – 0.014] | 0.53 |  |  |  |  |
|  | cg06825478 | chr6 | 31546067 | 3'UTR | -0.025 [-0.090 – 0.040] | 0.69 |  |  |  |  |

^a^ Base pair position of the CpG site within the genome (human genome 19).

^b^ A CpG site can be located within multiple genes or splice variants and thus can be situated within multiple gene sections.

^c^ Promoter is defined as 0 to 1500 base pairs upstream of the transcription start site.

^d^  Log fold change in M-values between former PICU patients and healthy controls adjusted for risk factors with corresponding 95% confidence interval.

^e^ p-values extracted from multivariable linear regression models built using the limma framework, adjusted for baseline risk factors and technical variation, and for multiple testing using a false discovery rate smaller than or equal to 0.05. All p-values come from separate models.

^f^ Classifications from the Functional Annotation of the Mammalian Genome (FANTOM) consortium as a low- or high-CpG density region associated with FANTOM4 promoters.

^g^ Chromosomal map coordinates from the FANTOM consortium of enhancer regions associated with FANTOM5 promoters.

^h^ Chromosomal map coordinates of the regulatory feature (informatically determined by the ENCODE Consortium).

^i^ Name of Differentially methylated region (DMR) in which these CpG sites were located (see DMR analysis: Supplementary Table S2).

Abbreviations: DMR: Differentially methylated region, Chr: Chromosome, PICU: paediatric intensive care unit, UTR: untranslated region.

**Table A2: DMR analysis – Former PICU patients vs Healthy children**

| **DMR** | **Gene name** | **Location ^a^** | | | **Width ^b^** | **Number of CpGs** | **Difference ^c^** | | |
| --- | --- | --- | --- | --- | --- | --- | --- | --- | --- |
|  |  | **Chr** | **Start** | **End** |  |  | **Maximum ^d^** | **Mean ^e^** | **p-value: Fisher ^f^** |
| DMR_1 | TNF | chr6 | 31543219 | 31543686 | 468 | 12 | -0.010 | -0.004 | 0.0041 |
| DMR_2 | TSC22D3 | chrX | 106959609 | 106960273 | 665 | 8 | 0.009 | 0.006 | 0.0072 |
| DMR_3 | AVP | chr20 | 3065343 | 3065488 | 146 | 4 | -0.014 | -0.011 | 0.0077 |

^a^ Chromosomal map coordinates of the DMR within human genome 19. DMRs were identified with the DMRcate package (Supplementary Method S3).

^b^ Width in number of base pairs between first and last CpG site within the DMR.

^c^ Difference between former PICU patients and Healthy children.

^d^ Maximum difference (expressed in M-values) between former patients and healthy children across all the CpG sites that are located within the DMR.

^e^ Mean difference (expressed in M-values) between former patients and healthy children across all the CpG sites that are located within the DMR.

^f^ p-value expressed as a Fisher combination of all the p-values across all the CpG sites that are located within the DMR.

Abbreviations: DMR: Differentially methylated region, Chr: Chromosome, PICU: paediatric intensive care unit.

**Table A3: DMP analysis – Former PICU patients vs Healthy children: Interaction with sex**

| **Gene name** | **CpG** | **Chr** | **Position ^a^** | **Gene section ^b, c^** | **Log Fold Change**  **[Confidence Interval] ^d^** | **p-value ^e^** | **FANTOM4**  **Enhancer location ^f^** | **FANTOM5**  **Enhancer location ^g^** | **ENCODE Regulatory Feature location ^h^** |
| --- | --- | --- | --- | --- | --- | --- | --- | --- | --- |
| CRHR1 | cg15607306 | chr17 | 43883843 | Body | 0.007 [-0.074 – 0.087] | 0.87 |  |  |  |
| POMC | cg09672383 | chr2 | 25390540 | 5'UTR | 0.020 [-0.080 – 0.120] | 0.70 |  |  |  |
| MC2R | cg26344168 | chr18 | 13884969 | Body | 0.032 [-0.049 – 0.114] | 0.44 |  |  |  |
| FKBP5 | cg22363520 | chr6 | 35558488 | Body | 0.018 [-0.145 – 0.182] | 0.83 |  |  |  |
|  | cg01839003 | chr6 | 35631009 | 5'UTR | 0.042 [-0.050 – 0.133] | 0.37 |  |  |  |
|  | cg03546163 | chr6 | 35654363 | 5'UTR | 0.003 [-0.131 – 0.136] | 0.97 |  |  |  |
|  | cg20813374 | chr6 | 35657180 | Promoter/5'UTR | 0.006 [-0.103 – 0.115] | 0.91 |  |  | 6:35655414-35657286 |
|  | cg03591753 | chr6 | 35659141 | 5'UTR | 0.024 [-0.060 – 0.108] | 0.58 |  |  |  |
|  | cg15929276 | chr6 | 35687457 | 5'UTR | -0.129 [-0.405 – 0.147] | 0.36 |  |  | 6:35687313-35687891 |
|  | cg23416081 | chr6 | 35693573 | 5'UTR | -0.083 [-0.351 – 0.184] | 0.54 |  | chr6:35693475-35693475 |  |
| NR3C1 | cg15910486 | chr5 | 142783621 | Promoter/5'UTR | 0.023 [-0.074 – 0.121] | 0.64 | high-CpG:142763715-142763897 |  | 5:142783021-142785530 |
|  | cg01967637 | chr5 | 142784019 | Promoter/5'UTR/1stExon | 0.060 [-0.061 – 0.18] | 0.33 | high-CpG:142763995-142764317 |  | 5:142783021-142785530 |
|  | cg26720913 | chr5 | 142814934 | 5'UTR/1stExon | -0.073 [-0.266 – 0.12] | 0.46 |  |  | 5:142813985-142815209 |
| HSD11B1 | cg14139038 | chr1 | 209877340 | Promoter/5'UTR | -0.031 [-0.106 – 0.045] | 0.42 |  |  |  |
|  | cg06571187 | chr1 | 209878123 | Promoter/5'UTR | 0.005 [-0.127 – 0.137] | 0.94 |  |  |  |
| SRD5A1 | cg22911074 | chr5 | 6632973 | Promoter | -0.017 [-0.112 – 0.077] | 0.72 |  |  | 5:6632952-6633655 |
| AKR1D1 | cg09373725 | chr7 | 137774429 | Body | -0.014 [-0.129 – 0.101] | 0.82 |  |  |  |
|  | cg27250318 | chr7 | 137775797 | Body | -0.040 [-0.248 – 0.168] | 0.70 |  |  |  |
|  | cg05082563 | chr7 | 137775882 | Body | -0.019 [-0.174 – 0.136] | 0.81 |  |  |  |
|  | cg02118020 | chr7 | 137782462 | Body | 0.135 [-0.096 – 0.366] | 0.25 |  |  |  |
| DUSP1 | cg00593243 | chr5 | 172196153 | Body | 0.118 [-0.097 – 0.333] | 0.28 |  |  | 5:172196108-172196195 |
| TSC22D3 | cg11907074 | chrX | 106959895 | Promoter/Body | -0.011 [-0.128 – 0.107] | 0.86 |  |  | X:106959379-106962296 |
|  | cg26954928 | chrX | 106978259 | Body | 0.053 [-0.066 – 0.171] | 0.38 |  |  |  |
| TNF | cg08553327 | chr6 | 31543647 | 1stExon | -0.005 [-0.076 – 0.066] | 0.89 |  |  | 6:31543103-31543774 |
|  | cg26729380 | chr6 | 31543655 | 1stExon | 0.010 [-0.052 – 0.073] | 0.75 |  |  | 6:31543103-31543774 |
|  | cg10650821 | chr6 | 31543686 | 1stExon | -0.007 [-0.065 – 0.052] | 0.82 |  |  | 6:31543103-31543774 |

^a^ Base pair position of the CpG site within the genome (human genome 19).

^b^ A CpG site can be located within multiple genes or splice variants and thus can be situated within multiple gene sections.

^c^ Promoter is defined as 0 to 1500 base pairs upstream of the transcription start site.

^d^ Log fold change in M-values between former PICU patients and healthy controls adjusted for risk factors with corresponding 95% confidence interval.

^e^ p-values extracted from multivariable linear regression models built using the limma framework, adjusted for baseline risk factors and technical variation. All p-values come from separate models.

^f^ Classifications from the Functional Annotation of the Mammalian Genome (FANTOM) consortium as a low- or high-CpG density region associated with FANTOM4 promoters.

^g^ Chromosomal map coordinates from the FANTOM consortium of enhancer regions associated with FANTOM5 promoters.

^h^ Chromosomal map coordinates of the regulatory feature (informatically determined by the ENCODE Consortium).

Abbreviations: Chr: Chromosome, PICU: paediatric intensive care unit, UTR: untranslated region.

**Table A4: DMP analysis – Former PICU patients vs Healthy children: Interaction with age at exposure**

| **Gene name** | **CpG** | **Chr** | **Position ^a^** | **Gene section ^b, c^** | **Log Fold Change**  **[Confidence Interval] ^d^** | **p-value ^e^** | **FANTOM4**  **Enhancer location ^f^** | **FANTOM5**  **Enhancer location ^g^** | **ENCODE Regulatory Feature location ^h^** |
| --- | --- | --- | --- | --- | --- | --- | --- | --- | --- |
| CRHR1 | cg15607306 | chr17 | 43883843 | Body | 0.000 [-0.008 – 0.009] | 0.92 |  |  |  |
| POMC | cg09672383 | chr2 | 25390540 | 5'UTR | -0.003 [-0.013 – 0.008] | 0.63 |  |  |  |
| MC2R | cg26344168 | chr18 | 13884969 | Body | 0.003 [-0.006 – 0.011] | 0.52 |  |  |  |
| FKBP5 | cg22363520 | chr6 | 35558488 | Body | -0.014 [-0.032 – 0.003] | 0.10 |  |  |  |
|  | cg01839003 | chr6 | 35631009 | 5'UTR | -0.008 [-0.018 – 0.002] | 0.11 |  |  |  |
|  | cg03546163 | chr6 | 35654363 | 5'UTR | -0.025 [-0.039 – -0.011] | 0.00063 |  |  |  |
|  | cg20813374 | chr6 | 35657180 | Promoter/5'UTR | -0.011 [-0.023 – 0.000] | 0.055 |  |  | 6:35655414-35657286 |
|  | cg03591753 | chr6 | 35659141 | 5'UTR | -0.012 [-0.021 – -0.004] | 0.0060 |  |  |  |
|  | cg15929276 | chr6 | 35687457 | 5'UTR | -0.003 [-0.032 – 0.026] | 0.84 |  |  | 6:35687313-35687891 |
|  | cg23416081 | chr6 | 35693573 | 5'UTR | -0.010 [-0.039 – 0.018] | 0.48 |  | chr6:35693475-35693475 |  |
| NR3C1 | cg15910486 | chr5 | 142783621 | Promoter/5'UTR | 0.001 [-0.010 – 0.011] | 0.88 | high-CpG:142763715-142763897 |  | 5:142783021-142785530 |
|  | cg01967637 | chr5 | 142784019 | Promoter/5'UTR/1stExon | 0.001 [-0.011 – 0.014] | 0.85 | high-CpG:142763995-142764317 |  | 5:142783021-142785530 |
|  | cg26720913 | chr5 | 142814934 | 5'UTR/1stExon | -0.011 [-0.032 – 0.009] | 0.28 |  |  | 5:142813985-142815209 |
| HSD11B1 | cg14139038 | chr1 | 209877340 | Promoter/5'UTR | -0.013 [-0.021 – -0.005] | 0.0020 |  |  |  |
|  | cg06571187 | chr1 | 209878123 | Promoter/5'UTR | -0.016 [-0.030 – -0.002] | 0.027 |  |  |  |
| SRD5A1 | cg22911074 | chr5 | 6632973 | Promoter | -0.001 [-0.011 – 0.009] | 0.91 |  |  | 5:6632952-6633655 |
| AKR1D1 | cg09373725 | chr7 | 137774429 | Body | -0.008 [-0.020 – 0.005] | 0.22 |  |  |  |
|  | cg27250318 | chr7 | 137775797 | Body | -0.009 [-0.031 – 0.013] | 0.42 |  |  |  |
|  | cg05082563 | chr7 | 137775882 | Body | -0.003 [-0.020 – 0.013] | 0.68 |  |  |  |
|  | cg02118020 | chr7 | 137782462 | Body | -0.014 [-0.039 – 0.010] | 0.26 |  |  |  |
| DUSP1 | cg00593243 | chr5 | 172196153 | Body | 0.011 [-0.012 – 0.034] | 0.34 |  |  | 5:172196108-172196195 |
| TSC22D3 | cg11907074 | chrX | 106959895 | Promoter/Body | -0.004 [-0.016 – 0.009] | 0.56 |  |  | X:106959379-106962296 |
|  | cg26954928 | chrX | 106978259 | Body | -0.012 [-0.025 – 0.001] | 0.061 |  |  |  |
| TNF | cg08553327 | chr6 | 31543647 | 1stExon | 0.003 [-0.005 – 0.010] | 0.50 |  |  | 6:31543103-31543774 |
|  | cg26729380 | chr6 | 31543655 | 1stExon | 0.001 [-0.006 – 0.007] | 0.81 |  |  | 6:31543103-31543774 |
|  | cg10650821 | chr6 | 31543686 | 1stExon | -0.002 [-0.009 – 0.004] | 0.45 |  |  | 6:31543103-31543774 |

^a^ Base pair position of the CpG site within the genome (human genome 19).

^b^ A CpG site can be located within multiple genes or splice variants and thus can be situated within multiple gene sections.

^c^ Promoter is defined as 0 to 1500 base pairs upstream of the transcription start site.

^d^ Log fold change in M-values between former PICU patients and healthy controls adjusted for risk factors with corresponding 95% confidence interval.

^e^ p-values extracted from multivariable linear regression models built using the limma framework, adjusted for baseline risk factors and technical variation. All p-values come from separate models.

^f^ Classifications from the Functional Annotation of the Mammalian Genome (FANTOM) consortium as a low- or high-CpG density region associated with FANTOM4 promoters.

^g^ Chromosomal map coordinates from the FANTOM consortium of enhancer regions associated with FANTOM5 promoters.

^h^ Chromosomal map coordinates of the regulatory feature (informatically determined by the ENCODE Consortium).

Abbreviations: Chr: Chromosome, PICU: paediatric intensive care unit, UTR: untranslated region

**Table A5: DMP analysis – Former PICU patients who received GC treatment vs those who did not**

| **Gene name** | **CpG** | **Chr** | **Position ^a^** | **Gene section ^b, c^** | **Log Fold Change**  **[Confidence Interval] ^d^** | **P-value ^e^** | **FANTOM4**  **Enhancer location ^f^** | **FANTOM5**  **Enhancer location ^g^** | **ENCODE Regulatory Feature location ^h^** |
| --- | --- | --- | --- | --- | --- | --- | --- | --- | --- |
| CRHR1 | cg15607306 | chr17 | 43883843 | Body | -0.009 [-0.067 – 0.050] | 0.77 |  |  |  |
| POMC | cg09672383 | chr2 | 25390540 | 5'UTR | 0.058 [-0.015 – 0.131] | 0.12 |  |  |  |
| MC2R | cg26344168 | chr18 | 13884969 | Body | 0.006 [-0.052 – 0.065] | 0.83 |  |  |  |
| FKBP5 | cg22363520 | chr6 | 35558488 | Body | -0.108 [-0.223 – 0.008] | 0.069 |  |  |  |
|  | cg01839003 | chr6 | 35631009 | 5'UTR | -0.071 [-0.138 – -0.004] | 0.037 |  |  |  |
|  | cg03546163 | chr6 | 35654363 | 5'UTR | 0.005 [-0.094 – 0.104] | 0.93 |  |  |  |
|  | cg20813374 | chr6 | 35657180 | Promoter/5'UTR | 0.020 [-0.057 – 0.097] | 0.61 |  |  | 6:35655414-35657286 |
|  | cg03591753 | chr6 | 35659141 | 5'UTR | 0.034 [-0.026 – 0.094] | 0.26 |  |  |  |
|  | cg15929276 | chr6 | 35687457 | 5'UTR | 0.298 [0.093 – 0.504] | 0.0045 |  |  | 6:35687313-35687891 |
|  | cg23416081 | chr6 | 35693573 | 5'UTR | 0.230 [0.040 – 0.419] | 0.018 |  | chr6:35693475-35693475 |  |
| NR3C1 | cg15910486 | chr5 | 142783621 | Promoter/5'UTR | -0.016 [-0.081 – 0.049] | 0.63 | high-CpG:142763715-142763897 |  | 5:142783021-142785530 |
|  | cg01967637 | chr5 | 142784019 | Promoter/5'UTR/1stExon | -0.071 [-0.152 – 0.009] | 0.081 | high-CpG:142763995-142764317 |  | 5:142783021-142785530 |
|  | cg26720913 | chr5 | 142814934 | 5'UTR/1stExon | 0.104 [-0.036 – 0.244] | 0.15 |  |  | 5:142813985-142815209 |
| HSD11B1 | cg14139038 | chr1 | 209877340 | Promoter/5'UTR | -0.019 [-0.073 – 0.035] | 0.50 |  |  |  |
|  | cg06571187 | chr1 | 209878123 | Promoter/5'UTR | 0.063 [-0.029 – 0.156] | 0.18 |  |  |  |
| SRD5A1 | cg22911074 | chr5 | 6632973 | Promoter | -0.075 [-0.137 – -0.013] | 0.018 |  |  | 5:6632952-6633655 |
| AKR1D1 | cg09373725 | chr7 | 137774429 | Body | 0.032 [-0.052 – 0.116] | 0.46 |  |  |  |
|  | cg27250318 | chr7 | 137775797 | Body | 0.106 [-0.044 – 0.255] | 0.17 |  |  |  |
|  | cg05082563 | chr7 | 137775882 | Body | 0.090 [-0.023 – 0.204] | 0.12 |  |  |  |
|  | cg02118020 | chr7 | 137782462 | Body | -0.199 [-0.364 – -0.035] | 0.018 |  |  |  |
| DUSP1 | cg00593243 | chr5 | 172196153 | Body | -0.123 [-0.275 – 0.029] | 0.11 |  |  | 5:172196108-172196195 |
| TSC22D3 | cg11907074 | chrX | 106959895 | Promoter/Body | 0.017 [-0.067 – 0.100] | 0.70 |  |  | X:106959379-106962296 |
|  | cg26954928 | chrX | 106978259 | Body | 0.034 [-0.047 – 0.115] | 0.41 |  |  |  |
| TNF | cg08553327 | chr6 | 31543647 | 1stExon | 0.028 [-0.023 – 0.079] | 0.29 |  |  | 6:31543103-31543774 |
|  | cg26729380 | chr6 | 31543655 | 1stExon | 0.010 [-0.035 – 0.056] | 0.66 |  |  | 6:31543103-31543774 |
|  | cg10650821 | chr6 | 31543686 | 1stExon | 0.029 [-0.013 – 0.071] | 0.17 |  |  | 6:31543103-31543774 |

^a^ Base pair position of the CpG site within the genome (human genome 19).

^b^ A CpG site can be located within multiple genes or splice variants and thus can be situated within multiple gene sections.

^c^ Promoter is defined as 0 to 1500 base pairs upstream of the transcription start site.

^d^ Log fold change in M-values between former PICU patients and healthy controls adjusted for risk factors with corresponding 95% confidence interval.

^e^ p-values extracted from multivariable linear regression models built using the limma framework, adjusted for baseline risk factors and technical variation. All p-values come from separate models.

^f^ Classifications from the Functional Annotation of the Mammalian Genome (FANTOM) consortium as a low- or high-CpG density region associated with FANTOM4 promoters.

^g^ Chromosomal map coordinates from the FANTOM consortium of enhancer regions associated with FANTOM5 promoters.

^h^ Chromosomal map coordinates of the regulatory feature (informatically determined by the ENCODE Consortium).

Abbreviations: Chr: Chromosome, GC: Glucocorticoid, PICU: paediatric intensive care unit, UTR: untranslated region

# ADDITIONAL References

1. McEwen LM, O'Donnell KJ, McGill MG, Edgar RD, Jones MJ, MacIsaac JL, Lin DTS, Ramadori K, Morin A, Gladish N, Garg E, Unternaehrer E, Pokhvisneva I, Karnani N, Kee MZL, Klengel T, Adler NE, Barr RG, Letourneau N, Giesbrecht GF, Reynolds JN, Czamara D, Armstrong JM, Essex MJ, de Weerth C, Beijers R, Tollenaar MS, Bradley B, Jovanovic T, Ressler KJ, Steiner M, Entringer S, Wadhwa PD, Buss C, Bush NR, Binder EB, Boyce WT, Meaney MJ, Horvath S, Kobor MS. The PedBE clock accurately estimates DNA methylation age in pediatric buccal cells. Proc Natl Acad Sci U S A. 2020;117:23329-35.

2. Solomon O, Huen K, Yousefi P, Kupers LK, Gonzalez JR, Suderman M, Reese SE, Page CM, Gruzieva O, Rzehak P, Gao L, Bakulski KM, Novoloaca A, Allard C, Pappa I, Llambrich M, Vives M, Jima DD, Kvist T, Baccarelli A, White C, Rezwan FI, Sharp GC, Tindula G, Bergstrom A, Grote V, Dou JF, Isaevska E, Magnus MC, Corpeleijn E, Perron P, Jaddoe VWV, Nohr EA, Maitre L, Foraster M, Hoyo C, Haberg SE, Lahti J, DeMeo DL, Zhang H, Karmaus W, Kull I, Koletzko B, Feinberg JI, Gagliardi L, Bouchard L, Ramlau-Hansen CH, Tiemeier H, Santorelli G, Maguire RL, Czamara D, Litonjua AA, Langhendries JP, Plusquin M, Lepeule J, Binder EB, Verduci E, Dwyer T, Carracedo A, Ferre N, Eskenazi B, Kogevinas M, Nawrot TS, Munthe-Kaas MC, Herceg Z, Relton C, Melen E, Gruszfeld D, Breton C, Fallin MD, Ghantous A, Nystad W, Heude B, Snieder H, Hivert MF, Felix JF, Sorensen TIA, Bustamante M, Murphy SK, Raikkonen K, Oken E, Holloway JW, Arshad SH, London SJ, Holland N. Meta-analysis of epigenome-wide association studies in newborns and children show widespread sex differences in blood DNA methylation. Mutat Res Rev Mutat Res. 2022;789:108415.

3. Kader F, Ghai M. DNA methylation-based variation between human populations. Mol Genet Genomics. 2017;292:5-35.

4. Aroke EN, Joseph PV, Roy A, Overstreet DS, Tollefsbol TO, Vance DE, Goodin BR. Could epigenetics help explain racial disparities in chronic pain? J Pain Res. 2019;12:701-10.

5. Raffington L, Tanksley PT, Sabhlok A, Vinnik L, Mallard T, King LS, Goosby B, Harden KP, Tucker-Drob EM. Socially stratified epigenetic profiles are associated with cognitive functioning in children and adolescents. Psychol Sci. 2023;34:170-85.

6. Panditharatna E, Filbin MG. The growing role of epigenetics in childhood cancers. Curr Opin Pediatr. 2020;32:67-75.

7. Gaal Z. Targeted epigenetic interventions in cancer with an emphasis on pediatric malignancies. Biomolecules. 2022;13:61.

8. Xie P, Zang LQ, Li XK, Shu Q. An epigenetic view of developmental diseases: new targets, new therapies. World J Pediatr. 2016;12:291-7.

9. Rangasamy S, D'Mello SR, Narayanan V. Epigenetics, autism spectrum, and neurodevelopmental disorders. Neurotherapeutics. 2013;10:742-56.

10. Xu K, Li S, Muskens IS, Elliott N, Myint SS, Pandey P, Hansen HM, Morimoto LM, Kang AY, Ma X, Metayer C, Mueller BA, Roberts I, Walsh KM, Horvath S, Wiemels JL, de Smith AJ. Accelerated epigenetic aging in newborns with Down syndrome. Aging Cell. 2022;21:e13652.

11. Lillycrop KA, Burdge GC. Epigenetic mechanisms linking early nutrition to long term health. Best Pract Res Clin Endocrinol Metab. 2012;26:667-76.

12. Gut P, Verdin E. The nexus of chromatin regulation and intermediary metabolism. Nature. 2013;502:489-98.

13. Peter CJ, Fischer LK, Kundakovic M, Garg P, Jakovcevski M, Dincer A, Amaral AC, Ginns EI, Galdzicka M, Bryce CP, Ratner C, Waber DP, Mokler D, Medford G, Champagne FA, Rosene DL, McGaughy JA, Sharp AJ, Galler JR, Akbarian S. DNA methylation signatures of early childhood malnutrition associated with impairments in attention and cognition. Biol Psychiatry. 2016;80:765-74.

14. Perfilyev A, Dahlman I, Gillberg L, Rosqvist F, Iggman D, Volkov P, Nilsson E, Risérus U, Ling C. Impact of polyunsaturated and saturated fat overfeeding on the DNA-methylation pattern in human adipose tissue: A randomized controlled trial. Am J Clin Nutr. 2017;105:991-1000.

15. Gur RE, Gur RC. Sex differences in brain and behavior in adolescence: Findings from the Philadelphia Neurodevelopmental Cohort. Neurosci Biobehav Rev. 2016;70:159-170.

16. Taddei M, Tinelli F, Faccio F, Riva D, Bulgheroni S. Sex influences on the neurocognitive outcome of preterm children. J Neurosci Res. 2023;101:796-811.

17. Jaber L, Halpern GJ, Shohat M. The impact of consanguinity worldwide. Community Genet. 1998;1:12-7.

18. Yengo L, Zhu Z, Wray NR, Weir BS, Yang J, Robinson MR, Visscher PM. Detection and quantification of inbreeding depression for complex traits from SNP data. Proc Natl Acad Sci U S A. 2017;114:8602-7.

19. Campbell LK, Scaduto M, Sharp W, Dufton L, Van Slyke D, Whitlock JA, Compas B. A meta-analysis of the neurocognitive sequelae of treatment for childhood acute lymphocytic leukemia. Pediatr Blood Cancer. 2007;49:65-73.

20. Gotte M, Kesting SV, Winter CC, Rosenbaum D, Boos J. Motor performance in children and adolescents with cancer at the end of acute treatment phase. Eur J Pediatr. 2015;174:791-9.

21. Nielsen MKF, Christensen JF, Frandsen TL, Thorsteinsson T, Andersen LB, Christensen KB, Nersting J, Faber M, Schmiegelow K, Larsen HB. Testing physical function in children undergoing intense cancer treatment-a RESPECT feasibility study. Pediatr Blood Cancer. 2018;65:e27100.

22. Yildiz Kabak V, Ipek F, Unal S, Atasavun Uysal S, Duger T. An evaluation of participation restrictions and associated factors via the ICF-CY framework in children with acute lymphoblastic leukemia receiving maintenance chemotherapy. Eur J Pediatr. 2021;180:1081-8.

23. Nyaradi A, Li J, Hickling S, Foster J, Oddy WH. The role of nutrition in children's neurocognitive development, from pregnancy through childhood. Front Hum Neurosci. 2013;7:97.

24. Burkhalter TM, Hillman CH. A narrative review of physical activity, nutrition, and obesity to cognition and scholastic performance across the human lifespan. Adv Nutr. 2011;2:201S-6S.

25. Anjos T, Altmae S, Emmett P, Tiemeier H, Closa-Monasterolo R, Luque V, Wiseman S, Perez-Garcia M, Lattka E, Demmelmair H, Egan B, Straub N, Szajewska H, Evans J, Horton C, Paus T, Isaacs E, van Klinken JW, Koletzko B, Campoy C, Group NR. Nutrition and neurodevelopment in children: focus on NUTRIMENTHE project. Eur J Nutr. 2013;52:1825-42.

26. Peters TJ, Buckley MJ, Statham AL, Pidsley R, Samaras K, Lord RV, Clark SJ, Molloy PL. De novo identification of differentially methylated regions in the human genome. Epigenetics Chromatin. 2015;8:6.

27. Mesotten D, Gielen M, Sterken C, Claessens K, Hermans G, Vlasselaers D, Lemiere J, Lagae L, Gewillig M, Eyskens B, Vanhorebeek I, Wouters PJ, Van Den Berghe G. Neurocognitive development of children 4 years after critical illness and treatment with tight glucose control: A randomized controlled trial. J Am Med Assoc. 2012;308:1641-50.

28. Van der Heijden KB, Suurland J, De Sonneville LM, Swaab H. BRIEF-P Vragenlijst voor executieve functies voor 2- tot 5-jarigen: Handleiding. Hogrefe; 2013.

29. Huizinga M, Smidts D. BRIEF Vragenlijst executieve functies voor 5- tot 18-jarigen: Handleiding. . Hogrefe; 2012.

30. Achenbach TM, Rescorla L, psychologiques Idr. Manual for the ASEBA Preschool Forms & Profiles: An Integrated System of Multi-informant Assessment. ASEBA; 2000.

31. Verhulst FC, Van der Ende J. Vragenlijsten voor leeftijden 6 tot en met 18 jaar [ASEBA Manual Questionnaires for ages 6 to 18 years]. ASEBA Nederland; 2013.

32. Hendriksen J, Hurks P. WPPSI-III-NL. Wechsler Preschool and Primary Scale of Intelligence: Handleiding. Pearson; 2010.

33. Wechsler D. WISC-III Nederlanstalige bewerking. Handleiding. Pearson; 2005.

34. Wechsler D. WAIS-III Nederlandstalige bewerking. Afname en Scoringshandleiding. Pearson; 2012.

35. Beery KE, Buktenica NA, Beery NA. The Beery-Buktenica Developmental Test of Visual-Motor Integration, 6th Edition (BEERY™ VMI). . Pearson; 2010.

36. De Sonneville L. Handboek Amsterdamse Neuropsychologische Taken. Sonares BV; 2014.

37. Cohen MJ. Children Memory Scale Manual. Pearson; 1997.
